# On Achievable Distortion in Sending Gaussian Sources over a Bandwidth-Matched Gaussian MAC with No Transmitter CSI

**DOI:** 10.3390/e21100992

**Published:** 2019-10-11

**Authors:** Chathura Illangakoon, Pradeepa Yahampath

**Affiliations:** Department of ECE, University of Manitoba, Winnipeg, MB R3T 5V6, Canada

**Keywords:** Gaussian sources, multiple-access channel, Rayleigh fading, channel-state information, joint source-channel coding, uncoded transmission

## Abstract

This paper investigates the minimum mean square error (MMSE) of communicating a pair of Gaussian sources over a a bandwidth-matched Gaussian multiple-access channel with block Rayleigh fading in the absence of channel state information (CSI) at the transmitters. The achievable MMSE is not known. To this end, we derive several upper-bounds to the minimum achievable average MMSE as a function of the transmitter powers, the average channel fading power-to-noise ratio, and the correlation coefficient of the two sources. To derive nontrivial upper bounds which improve on those of separate source-channel coding and uncoded transmission, we incorporate ideas from joint source-channel coding and hybrid digital–analog coding to construct specific coding schemes for which the achievable MMSE can be determined.

## 1. Introduction

An important problem in wireless communication is the design of systems that are robust against random variations in the channel signal-to-noise ratio (CSNR) caused by fading. If the channel response can be measured at both transmitter and receiver prior to transmitting each codeword and the channel remains stationary during the transmission of a codeword, adaptive transmitters and receivers can be used to achieve optimal communication. Although the receiver adaptation is feasible in most cases, the transmitter adaptation can be impractical in some cases. An obvious case is a single transmitter communicating with multiple receivers over a broadcast channel (BC). Another case is multiple transmitters communicating with a common receiver over a multiple-access channel (MAC) where the individual transmitters have no access to the respective CSNRs observed at the receiver. An important practical application of the latter case is a wireless sensor network (WSN) [1], where possibly correlated, sampled analog signals sensed at multiple locations are transmitted to a single receiver over a MAC [2]. The work presented in this paper is an attempt to characterize the theoretical limits to performance achievable in transmitting a pair of sampled Gaussian sources over a Gaussian MAC (GMAC) to a common receiver. More specifically, the basic question of interest is, “what is the total minimum mean square error (MMSE) with which we can reconstruct at a common receiver, a pair of Gaussian sources transmitted over a two-user power-limited GMAC with block fading, when the receiver knows the channel state information (CSI) but the transmitters only have prior knowledge of the distribution of CSI?” The problem involves averaging the achievable MMSE for a given channel state over the CSI distribution. A complete answer to this question remains an open problem. In this paper, we partially answer this question by considering certain coding schemes for which the MMSE can be computed. The answer to our question partly depends on whether or not the two sources are correlated. Both cases are considered in this paper. We limit attention to the particular case where transmission rate is one source sample per channel use, or in other words, the bandwidth of each source is identical to the GMAC bandwidth (“bandwidth-matched”).

### 1.1. Related Work

Asymptotically optimal (achieves the MMSE as the codeword length approaches infinity) communication of a Gaussian source over a point-to-point block fading channel whose CSI is known to both the transmitter and the receiver can be achieved by separate source-channel (SSC) coding, i.e., by cascading an optimal vector quantizer (VQ) for the Gaussian source with a capacity achieving channel code for the Gaussian channel [3]. Even if CSI is available at the transmitters, the source-channel separation is not in general optimal for communication over MACs ([4], Ch. 15) [5]. General conditions under which the optimality of separation holds for Gaussian sources and a GMAC are not completely known. Some special cases are however known. It is known that separation is optimal for orthogonalized transmission over a GMAC if the CSI is available at both the transmitters and the receiver [6]. When the sources are memoryless and mutually independent, SSC coding is also known to be optimal for the so-called two-to-one GMAC with no fading (NF-GMAC) [7]. In both cases, the MMSEs achievable for a set of Gaussian sources at given rates can be obtained by combining the rate-distortion functions of the sources and the capacity region of the GMAC. For a block-fading MAC (BF-MAC), the same optimality result applies if the CSI is available at both the transmitters and the receiver. In this case, the optimality can be achieved by adaptive coding at each transmitter. This is, however, not possible if CSI is not available at the transmitters.

For the transmission of mutually correlated Gaussian sources over a NF-GMAC, SSC coding requires, at each transmitter, a cascade of an optimal distributed VQ [8] and a capacity achieving channel code for the GMAC. It is, however, known that this approach is not optimal even if the transmitters know CSI [9]. This follows from a simple observation regarding the channel capacity. When the sources at the inputs are correlated, the maximum mutual information between the inputs and the output of a two-to-one MAC can be made higher than that with uncorrelated inputs, which implies that the achievable rate region of a MAC for correlated sources is larger than that for uncorrelated sources. However, realizing the rates in the enlarged region necessitates joint source-channel (JSC) codes capable of creating mutually correlated inputs to the GMAC. For example, if the source sequences themselves are used as the channel codewords, the source correlation is directly transferred to the GMAC inputs. This the simplest possible JSC coding scheme and is commonly referred to as “uncoded” or “amplify-and-forward” transmission. Despite the simplicity of this scheme, it is shown in [10] that uncoded transmission is optimal for transmitting two memoryless and correlated Gaussian sources with equal bandwidths over a two-to-one NF-GMAC with the same bandwidth, if the CSNR is below a threshold that is determined by the correlation coefficient between the two sources. Furthermore, the MMSE of uncoded transmission remains below that of SSC coding for a wide range of CSNRs even above this threshold. This is in sharp contrast to orthogonal multiple access over a NF-GMAC, in which the separation is strictly optimal at all CSNRs [6]. However, as the CSNR approaches infinity, SSC coding on a two-to-one NF-GMAC outperforms uncoded transmission. Intuitively, if the MAC is noisy, the dependence between the channel inputs allows better estimation of the individual inputs from their noisy sum observed at the channel output; whereas, if the MAC is almost noise-free, there is little to be gained by having dependent channel inputs. In the latter case, proper coding allows recovering the individual GMAC inputs error-free, from which each source can be reconstructed within the quantization error. This is not possible when the GMAC output is the direct sum of the two sources as in the case of uncoded transmission. Another instance of the NF-GMAC in which the uncoded transmission is optimal is the so-called “CEO problem” [11]. In the simplest instance of the CEO problem, the transmitters at the inputs of a GMAC observe noisy versions of the same memoryless Gaussian source, and the objective is to estimate the this source from the GMAC output. For this set-up, if the sources and the channel are bandwidth matched, the uncoded transmission is optimal regardless of the CSNR [12], whereas the SSC coding is suboptimal. This result is not surprising if one considers the fact that, for a single memoryless Gaussian source and a memoryless Gaussian channel with identical bandwidths, the uncoded transmission is optimal [13].

The trade-off between the energy per channel use and the achievable distortion in transmission of two correlated Gaussian sources over a NF-GMAC is studied in [14]. The focus in [14] is the minimum transmit energy pairs, which can achieve a given distortion pair with no restriction on the source-channel bandwidth ratio. Interestingly, the analysis in [14] reveals that, with no feedback from the receiver to the transmitters, uncoded transmission is more energy efficient than SSC coding for sufficiently large distortion targets.

### 1.2. Main Contributions

This paper derives a number of upper-bounds to the average MMSE, referred to as the fading-averaged MMSE (FA-MMSE), achievable in sending a pair of Gaussian sources over a GMAC with Rayleigh-fading and no transmit-side CSI, as a function of transmitter powers, average channel fading power-to-noise ratio, and source correlation coefficient. We refer to the MMSE in this case as the distortion power function (DPF) for Gaussian sources and GMAC. What is derived here are the upper bounds of the unknown DPF.

1. The obvious and relatively straightforward to-determine upper-bounds to the DPF are the FA-MMSEs, which are achievable with uncoded transmission and SSC coding. In this paper, we derive an alternative bound by considering conventional hybrid digital–analog (HDA) coding, wherein vector quantization error in conventional digital coding is transmitted in analog form by superposition. As expected, the numerical results show that, for uncorrelated sources, HDA coding improves on both SSC coding and uncoded transmission. For correlated sources, uncoded transmission has an advantage over HDA coding at low CSNRs where digital coding frequently suffers receiver outages due to lack of CSI at the the transmitters.
2. It is shown in [10] that when the sources are correlated and the GMAC is fixed (no fading), although uncoded transmission of the sources over the GMAC is optimal at SNRs below a threshold determine by the source correlation, the uncoded transmission of vector quantized sources directly over the GMAC (JSC-VQ) is asymptotically (as the CSNR approaches infinity) optimal. Furthermore, it has been shown that this scheme, when enhanced with a superimposed uncoded transmission of the sources (HDA-JSC-VQ), is nearly optimal at all CSNRs. Based on these observations, we derive two upper bounds to the DPF for the fading GMAC, referred as the JSC-VQ bound and HDA-JSC-VQ bound, respectively. Although these bound do not have expressions that can be readily interpreted, they can be numerically computed. It is observed that JSC-VQ and HDA-JSC-VQ bounds are not significantly different, regardless of the source correlation. However, the comparison of these bounds with the distortion bound for SSC coding shows a gap that grows with source correlation and CSNR. Although there exists a gap even when the sources are uncorrelated, this gap is relatively much smaller. The HDA-JSC-VQ bound established here is the lowest known upper bound to unknown DPF. It is shown that, for highly correlated sources and under low average CSNRs, uncoded transmission can achieve performance approaching the HDA-JSC-VQ bound.

## 2. Problem Definition

We begin with a formal statement of the basic problem addressed in this paper. Suppose we observe two continuous-valued information sources, $S_{1}$ and $S_{2}$, at different locations and there is no communication link between the two locations. We wish to communicate and reproduce these two sources at a central location, where the communication takes place over a wireless channel modeled by a two-to-one GMAC with block Rayleigh fading (BF-GMAC). The fading gains of the GMAC are known to the receiver, but are not known to the the respective transmitters. Each source is a circularly symmetric complex-valued Gaussian variable, $S_{i}\in C$, with mean zero, variance of $E\{|S_{i}{|}^{2}\}=\sigma^{2}$, and the correlation $E\left\{S_{1}^{*}S_{2}\right\}=\rho \sigma^{2}$, where |ρ|<1. A sequence of *n* samples from the source $S_{i}$, denoted by $S_{i}=\left(S_{i,1},\ldots ,S_{i,n}\right)$, is assumed to be independent and identically distributed (iid). Our interest in this paper is the transmission of a sequence of *n* source samples in *n* uses of the GMAC. The encoder for source $S_{i}$ is therefore a mapping $f_{i}^{\left(n\right)}:C^{n}\to C^{n}$, where the channel codeword $X_{i}=\left(X_{i,1},\ldots ,X_{i,n}\right)$ is given by

$$X_{i}=f_{i}^{\left(n\right)}\left(S_{i}\right),$$

and $X_{i,k}\in C$ is the channel input for $S_{i}$ at time k=1,…,n (the superscript in $f_{i}^{\left(n\right)}$ emphasizes the fact that each encoder is a block-encoder for *n* connective source samples.) The transmitter for $S_{i}$ has an average power constraint, $P_{i}$, so that

(1) $$\begin{array}{c} \frac{1}{n}\sum_{k=1}^{n}E\left\{|X_{i,k}{|}^{2}\right\}\leq P_{i}, \end{array}$$

i=1,2.

Let the GMAC output for the input codeword pair $\left(X_{1},X_{2}\right)$ be the sequence $Y=(Y_{1},\ldots ,Y_{n})$, where $Y_{k}\in C$ is the GMAC output at time *k* given by

$$Y_{k}=h_{1,k}X_{1,k}+h_{2,k}X_{2,k}+W_{k},$$

$h_{i,k}\in C$ is the gain of the channel between $S_{i}$ and the receiver at time *k* and $W_{k}\in C$ is complex-valued channel noise. As usual, it is assumed that $\left(h_{1,k},h_{2,k}\right)$ are iid complex Gaussian random variables with mean zero and independent real and complex parts. In a BF-GMAC, $h_{1,k}$ and $h_{2,k}$ remain constant during the transmission of a length *n* codeword. Therefore, henceforth we will drop the time index, *k*, denote the channel gains by $h_{1}$ and $h_{2}$, and denote $\left(h_{1},h_{2}\right)$ by h. The channel noise, $W_{k}$, is assumed to be circularly symmetric Gaussian random variable with mean zero and variance *N*. The noise $W=(W_{1},\ldots W_{n})$ is assumed to be an iid sequence. For convenience, define the CSNR $\Gamma_{i}={\left|h_{i}\right|}^{2}/N$ and $\gamma_{i}={\left|h_{i}\right|}^{2}$, which is the exponentially distributed power gain of the channel i=1,2. Let $E\left\{\gamma_{i}\right\}=\bar{\gamma }$. The total output CSNR in the channel state h is

$$\Gamma =\frac{\gamma_{1}P_{1}+\gamma_{2}P_{2}+2\rho_{x}\gamma_{12}\sqrt{P_{1}P_{2}}}{N}=\Gamma_{1}P_{1}+\Gamma_{2}P_{2}+2\rho_{x}\Gamma_{12}\sqrt{P_{1}P_{2}},$$

where $\gamma_{12}=Re\left\{h_{1}h_{2}^{*}\right\}$, $\rho_{x}=\frac{E\{X_{1}X_{2}^{*}\}}{\sqrt{P_{1}P_{2}}}$, and $\Gamma_{12}=\gamma_{12}/N$. We will refer to $\bar{\Gamma }=\frac{\bar{\gamma }}{N}$ as the “fading power-to-noise ratio” (FPNR) of the channel, which is a figure-of-merit for the BF-GMAC. Note that $\rho_{x}$ depends on the encoding scheme. For example, if SSC coding is used, the GMAC inputs are independent and we will have $\rho_{x}=0$ regardless of source correlation ρ. On the other hand, if uncoded transmission is used, $\rho_{x}=\rho$.

The receiver observes the channel output Y and the channel state $h=(h_{1},h_{2})$ and reconstructs the sequences $S_{1}$ and $S_{2}$. This decoder can be described by a pair of mappings $\phi_{i}^{\left(i\right)}:C^{n}\times C^{2}\to C^{n}$, such that the decoded source sequences are given by

$${\hat{S}}_{i}=\phi_{i}^{\left(n\right)}\left(Y,h\right),\;i=1,2.$$

We will measure the distortion between $S_{i}$ and ${\hat{S}}_{i}$ using the average MSE, given by

$$d_{i}=\frac{1}{n}\sum_{k=1}^{n}E{\left|S_{i,k}-{\hat{S}}_{i,k}\right|}^{2},$$

For notational simplicity, we denote the minimum achievable $d_{i}$ for a fixed channel state h by $d_{i}\left(h\right)$ and let

$$d_{1,2}\left(h\right)=d_{1}\left(h\right)+d_{2}\left(h\right).$$

Our main goal in this paper is to determine the distortion power function (DPF) for two Gaussian sources and a BF-GMAC, given by

(2) $$D\left(P_{1},P_{2}\right)=\underset{f_{1},f_{2},\phi_{1},\phi_{2}}{\inf}\frac{1}{2}\int d_{1,2}\left(h\right)p\left(h\right)dh,$$

where $p\left(h\right)=p_{1}\left(h_{1}\right)p_{2}\left(h_{2}\right)$ and $p_{i}\left(h\right)$ is the pdf of $h_{i}$, i=1,2. Note that $D(P_{1},P_{2})$ is defined for a given source correlation ρ and a FPNR $\bar{\Gamma }$ of a bandwidth-matched BF-GMAC. An alternative description of $D(P_{1},P_{2})$ is the achievable power region $\left(P_{1},P_{2}\right)$ for a given target *D*. Note that DPF for a single Gaussian source and a point-point AWGN channel can be found by evaluating the distortion rate function of the source at the rate equal to the channel capacity, i.e., $D\left(P\right)=\sigma^{2}2^{-\log_{2}\left(1+P/N\right)}$ [4].

Finding $D(P_{1},P_{2})$ in general is difficult. Therefore, our end goal is to find useful upper-bounds to $D(P_{1},P_{2})$, by considering certain coding schemes for which $d_{1}\left(h\right)$ and $d_{2}\left(h\right)$ can be found in closed-form, and therefore Equation (2) can be at least numerically evaluated.

### Notation and Terminology

For simplicity of presentation, throughout the paper we define the index variable i∈{1,2}. The index j∈{1,2} is always defined in relation to *i* as follows,

$$j=\left\{\begin{array}{cc} 2 & \;\mathrm{if}\;i=1\\ 1 & \;\mathrm{if}\;i=2. \end{array}\right.$$

The complex conjugate of *X* is denoted by $X^{*}$. The transpose and conjugate transpose (Hermitian) of a matrix X are denoted by $X^{T}$ and $X^{H}$, respectively. The time-averaged expectation of a length *n* sequence $X_{1},\ldots ,X_{n}$ will be denoted by

$$\begin{array}{c} \frac{1}{n}\sum_{k=1}^{n}E\left\{X_{k}\right\}={\bar{E\{X\}}}_{n}. \end{array}$$

## 3. Separate Source-Channel Coding

As a benchmark, we consider ubiquitous SSC coding. In this case, the encoder mapping $X_{i}=f_{i}^{\left(n\right)}\left(S_{i}\right)$ is a concatenation of two-stages. In the first, the sequence $S_{i}$ is vector-quantized to produce a “digital” index $I_{i}=\Pi_{i}^{\left(n\right)}\left(S_{i}\right)$. In the second stage, the index $I_{i}$ is encoded into a channel codeword $X_{i}=\Lambda_{i}^{\left(n\right)}\left(I_{i}\right)$. The first-stage (VQ) is a mapping $\Pi_{i}^{\left(n\right)}:C^{n}\to \left\{0,\ldots ,2^{nR_{i}}-1\right\}$, and the second stage (channel encoder) is a mapping $\Lambda_{i}^{\left(n\right)}:\left\{0,\ldots ,2^{nR_{i}}-1\right\}\to C^{n}$, where $R_{i}$ is the rate of the encoder *i* in bits/channel-use, i=1,2. If $S_{1}$ and $S_{2}$ are uncorrelated, $\Pi_{i}^{\left(n\right)}$ is a rate-distortion optimal VQ for $S_{i}$. If $S_{1}$ and $S_{2}$ are correlated, the pair $\left(\Pi_{1}^{\left(n\right)},\Pi_{2}^{\left(n\right)}\right)$ is an optimal distributed VQ for $\left(S_{1},S_{2}\right)$ [15]. The decoder $\phi_{i}^{\left(n\right)}$ also consists of two stages. The first stage (channel decoder) decodes the index $I_{i}$ using Y and h. As usual, we will say that a transmitted channel codeword is “correctly decodable”, or simply decodable, if the codeword can be recovered from the channel output with an arbitrarily small error probability by letting n→∞. The second stage (source decoder) optimally estimates $S_{i}$ using recovered $\left(I_{1},I_{2}\right)$, i.e., with MMSE estimation, ${\hat{S}}_{i}=E\left\{S_{i}|I_{1},I_{2}\right\}$. However, as the transmitters do not observe h, the source and channel codes cannot be chosen adaptively to guarantee the error-free recovery of $\left(I_{1},I_{2}\right)$. With fixed $f_{i}^{\left(n\right)}$, i=1,2, depending on the realization of h the received channel codewords may or may not be decodable. The event where only a single codeword, either $X_{1}$ or $X_{2}$, can be decoded is referred to as a “partial outage” and that where both codewords are undecodable is referred to as a “total outage”.

Let $E_{i}$ denote the event that $I_{i}$ is decoded correctly and $E_{12}$ denote the no-outage event that both codewords are decoded correctly (no-outage event). Further, let $E_{i}^{\prime }$ denote the partial-outage event that only the codeword $I_{i}$ is correctly decoded and $E_{12}^{\prime }$ denote the total-outage event. The probabilities of outage events for uncorrelated sources and transmitters with the same power have been determined in [16]. In general, $P(E|R_{1},R_{2})$, given $\left(R_{1},R_{2}\right)$, can be found as shown in Appendix A. Let $d(E|R_{1},R_{2})$ denote the conditional MMSE under the outage event E, given $\left(R_{1},R_{2}\right)$. If the conditional fading-averaged MMSE (FA-MMSE), given $\left(R_{1},R_{2}\right)$, is $\bar{d}\left(R_{1},R_{2}\right)$, then the minimum FA-MMSE achievable with SSC coding is

(3) $$\begin{array}{c} D^{SSC}\left(P_{1},P_{2}\right)=\min_{R_{1},R_{2}}\bar{d}\left(R_{1},R_{2}\right). \end{array}$$

### 3.1. Uncorrelated Sources

In this case, the reconstruction of $S_{i}$ only requires $I_{i}$ and straightforwardly

$$\bar{d}\left(R_{1},R_{2}\right)=\frac{\sigma^{2}}{2}\sum_{i=1}^{2}\left\{2^{-2R_{i}}Pr\left(E_{i}|R_{1},R_{2}\right)+\left[1-Pr\left(E_{i}|R_{1},R_{2}\right)\right]\right\}.$$

### 3.2. Correlated Sources

When the sources are correlated, the source encoders must constitute a distributed VQ. One issue with an optimal distributed VQ is that, due to the mutual dependence of quantizers for the two sources, the reconstruction of neither source is possible unless the channel codewords from both transmitters can be correctly decoded. Therefore,

$$\bar{d}\left(R_{1},R_{2}\right)=d\left(E_{12}|R_{1},R_{2}\right)p_{12}+\sigma^{2}\left(1-p_{12}\right),$$

where $p_{12}=Pr\left(E_{12}|R_{1},R_{2}\right)$ and $d(E_{12}|R_{1},R_{2})$ in this case are the minimum achievable MSE $D_{DVQ}\left(R_{1},R_{2}\right)$ of a distributed VQ with rates ($R_{1},R_{2})$, which is given by the the following lemma.

**Lemma** **1.** *Let*

$$\Delta \left(x,y\right)=2^{-2x}\left(1-\rho^{2}+\rho^{2}2^{-2y}\right)$$

*and $R_{sum}=R_{1}+R_{2}$. The MMSE of a distributed VQ for a pair of mean-zero, variance $\sigma^{2}$ Gaussian sources with the correlation coefficient ρ is given by*

$$D_{DVQ}\left(R_{1},R_{2}\right)=\sigma^{2}\left(\Delta^{*}+\frac{\Delta (R_{sum},R_{sum})}{\Delta^{*}}\right)$$

*where*

(4) $$\Delta^{*}=\max\left\{\Delta \left(R_{1},R_{2}\right),\Delta \left(R_{2},R_{1}\right),\sqrt{\Delta (R_{sum},R_{sum})}\right\}.$$

**Proof.**  See Appendix B. □

## 4. Conventional HDA Coding

Both fully analog (uncoded) transmission and fully digital SSC coding are special cases of more general HDA coding, where the total power and/or channel bandwidth are split between an analog encoder and a conventional digital encoder. When CSI is not available at the transmitters, an HDA system with a power allocation optimized for the channel state distribution can always outperform (in a FA-MMSE sense) both the uncoded and SSC-coded transmission. In this section, we analyze an HDA scheme that uses a conventional SSC encoder (optimal VQ in cascade with a channel coder for GMAC) as the digital part and the quantization error as the analog part [17]. The conventional approach to combining the analog and digital channel signals is by superposition as considered below. Later, in Section 5.1, we will consider an alternative approach where a vector quantizer is used a JSC code in the digital part.

**Figure 1:**
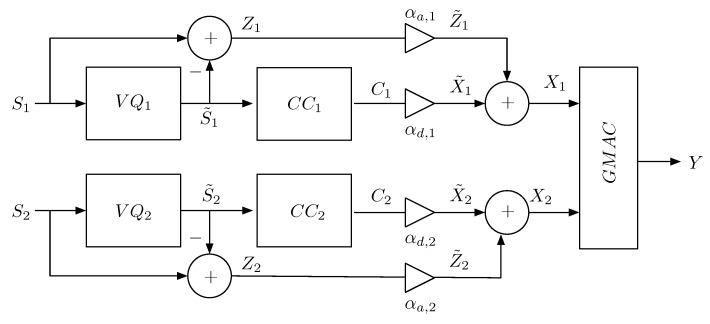
Hybrid digital–analog (HDA) transmission of Gaussian source over GMAC. VQ: vector quantizer; CC: channel encoder.

A conventional HDA system is shown in Figure 1. The analog and digital components of each transmitter output share the same channel bandwidth via superposition, whereas the total available transmitter power is split between the two components via digital and analog scaling factors $\left(\alpha_{di},\alpha_{ai}\right)$. The decoding at the GMAC output relies on the principle of successive interference cancellation (SIC). Each encoder is parameterized by $\left(R_{i},t_{i}\right)$, where $R_{i}$ is VQ rate and $0\leq t_{i}\leq 1$ is the digital–analog power allocation factor to be introduced below, i=1,2. Note that in this HDA system, each source encoder is a rate-distortion optimal VQ for the respective source, regardless of whether the sources are correlated or not. Therefore, unlike in Section 3, the source reconstruction becomes possible even under partial-outage events. Furthermore, if the sources are correlated, the quantization errors are also correlated (which is not the case if a distributed VQ is used), allowing analog components of the HDA transmission to interfere, on average, in a constructive manner over the GMAC.

Let the quantized value and quantization error for source sequence $S_{i}$ be ${\tilde{S}}_{i}$ and $Z_{i}$, respectively. For rate-distortion optimal VQ of a Gaussian source, the quantization error variance is [4]

$$\begin{array}{c} {\bar{E\{|Z_{i}{|}^{2}\}}}_{n}=\sigma_{z_{i}}^{2}=\sigma^{2}2^{-2R_{i}}, \end{array}$$

where $Z_{i}=\left(Z_{i,1},\ldots ,Z_{i,n}\right)$. Furthermore, ${\tilde{S}}_{i}$ and $Z_{i}$ are uncorrelated, and therefore

$$\begin{array}{c} {\bar{E\{|{\tilde{S}}_{i}{|}^{2}\}}}_{n}={\tilde{\sigma }}_{i}^{2}=\sigma^{2}\left(1-2^{-2R_{i}}\right). \end{array}$$

where ${\tilde{S}}_{i}=\left({\tilde{S}}_{i,1}\cdots ,{\tilde{S}}_{i,n}\right)$. For correlated Gaussian sources, the following results regarding the time-averaged asymptotic cross-correlations hold [10].

(5) $$\begin{array}{cc} {\bar{E\{S_{i}^{*}{\tilde{S}}_{i}\}}}_{n} & =\sigma^{2}\left(1-2^{-2R_{i}}\right) \end{array}$$

(6) $$\begin{array}{cc} {\bar{E\{S_{i}^{*}{\tilde{S}}_{j}\}}}_{n} & =\sigma^{2}\rho \left(1-2^{-2R_{j}}\right) \end{array}$$

(7) $$\begin{array}{cc} {\bar{E\{{\tilde{S}}_{1}^{*}{\tilde{S}}_{2}\}}}_{n} & =\sigma^{2}\rho \left(1-2^{-2R_{1}}\right)\left(1-2^{-2R_{2}}\right) \end{array}$$

(8) $$\begin{array}{cc} {\bar{E\{Z_{1}^{*}Z_{2}\}}}_{n} & =\rho \sigma^{2}2^{-2(R_{1}+R_{2})}. \end{array}$$

Further, we define the correlation coefficients

(9) $$\begin{array}{cc} \tilde{\rho } & =\frac{{\bar{E\{{\tilde{S}}_{1}^{*}{\tilde{S}}_{2}\}}}_{n}}{{\tilde{\sigma }}_{1}\tilde{\sigma_{2}}}=\rho \sqrt{\left(1-2^{-2R_{1}}\right)\left(1-2^{-2R_{2}}\right)}, \end{array}$$

(10) $$\begin{array}{cc} \rho_{z} & =\frac{{\bar{E\{Z_{1}^{*}Z_{2}\}}}_{n}}{\sigma_{z_{1}}\sigma_{z_{2}}}. \end{array}$$

The VQ codeword ${\tilde{S}}_{i}$ (specifically, an index identifying it) is encoded into a channel codeword $C_{i}$ of a capacity achieving channel code for the GMAC. The channel input for source sequence $S_{i}$ is given by

$$X_{i}={\tilde{X}}_{i}+{\tilde{Z}}_{i},$$

where ${\tilde{X}}_{i}=\alpha_{di}C_{i}$ and ${\tilde{Z}}_{i}=\alpha_{ai}Z_{i}$, and $0\leq \alpha_{di}\leq 1$ and $0\leq \alpha_{ai}\leq 1$ are chosen such that

$$\alpha_{di}^{2}{\bar{E\{|C_{i}{|}^{2}\}}}_{n}+\alpha_{ai}^{2}{\bar{E\{|Z_{i}{|}^{2}\}}}_{n}=P_{i},\;i=1,2.$$

We define the digital–analog power allocation factors as $t_{i}=\frac{\alpha_{ai}^{2}{\bar{E\{|Z_{i}{|}^{2}\}}}_{n}}{P_{i}}$, i=1,2, so that $\alpha_{di}^{2}{\bar{E\{|C_{i}{|}^{2}\}}}_{n}=\left(1-t_{i}\right)P_{i}$. The resulting GMAC output is given by

$$\begin{array}{c} Y_{k}=h_{1}\left({\tilde{X}}_{1,k}+{\tilde{Z}}_{1,k}\right)+h_{2}\left({\tilde{X}}_{2,k}+{\tilde{Z}}_{2,k}\right)+W_{k},\;k=1,\ldots ,n, \end{array}$$

where ${\tilde{X}}_{i}=\left({\tilde{X}}_{i,1},\ldots ,{\tilde{X}}_{i,n}\right)$, $W=(W_{1},\ldots ,W_{n})$ and $Y=(Y_{1},\ldots ,Y_{n})$.

The analog channel inputs act as noise to the digital channel decoder which jointly decodes the two codewords $C_{1}$ and $C_{2}$. Recall that with asymptotically optimal VQ, ${\tilde{Z}}_{i,k}$s are iid Gaussian variables and therefore the total noise ${\tilde{Z}}_{i}+W_{i}$ at the input of the channel decoder is also iid Gaussian. Digital codewords are decoded first and the correctly decoded codewords are then used to cancel out the digital channel inputs from the observed channel output. The source sequences are then linearly estimated from the correctly decoded channel codewords and the residual channel output. The achievable MMSE of the HDA system in any given channel state h depends on the the decodability of digital codewords $C_{1}$ and $C_{2}$. Let $d_{i}\left(E|R_{1},R_{2},t_{1},t_{2}\right)$ be the conditional MMSE for source *i* under the event E, given $R_{1}$, $R_{2}$, $t_{1}$, and $t_{2}$, i=1,2. The necessary and sufficient conditions for each event can be found using the basic achievable rate region for a GMAC [4].

- No outage event $E_{12}$: Both $C_{1}$ and $C_{2}$ are decodable if and only if ([4], Equations 15.147–15.149)
  (11) $$\begin{array}{cc} R_{i} & <\frac{1}{2}\log\left(1+\frac{\left(1-t_{i}\right)P_{i}\Gamma_{i}}{t_{i}P_{i}\Gamma_{i}+t_{j}P_{j}\Gamma_{j}+2\Gamma_{12}\rho_{z}\sqrt{t_{1}t_{2}P_{1}P_{2}}+1}\right),\;i=1,2 \end{array}$$
  (12) $$\begin{array}{cc} R_{1}+R_{2} & <\frac{1}{2}\log\left(1+\frac{\left(1-t_{1}\right)P_{1}\Gamma_{1}+\left(1-t_{2}P_{2}\right)\Gamma_{2}}{t_{1}P_{1}\Gamma_{1}+t_{2}P_{2}\Gamma_{2}+2\Gamma_{12}\rho_{z}\sqrt{t_{1}t_{2}P_{1}P_{2}}+1}\right). \end{array}$$
- Partial outage event $E_{i}^{\prime }$ (either $C_{1}$ or $C_{2}$ is decodable, but not both): $C_{i}$ is decodable while $C_{j}$ is not decodable if and only if
  (13) $$\begin{array}{cc} R_{i} & <\frac{1}{2}\log\left(1+\frac{\left(1-t_{i}\right)P_{i}\Gamma_{i}}{t_{1}P_{1}\Gamma_{1}+P_{2}\Gamma_{2}+2\Gamma_{12}\rho_{z}\sqrt{t_{1}t_{2}P_{1}P_{2}}+1}\right), \end{array}$$
  (14) $$\begin{array}{cc} R_{j} & >\frac{1}{2}\log\left(1+\frac{\left(1-t_{j}\right)P_{j}\Gamma_{j}}{t_{i}P_{i}\Gamma_{i}+t_{j}P_{j}\Gamma_{j}+2\Gamma_{12}\rho_{z}\sqrt{t_{1}t_{2}P_{1}P_{2}}+1}\right), \end{array}$$
- Total outage event $E_{12}^{\prime }$: Neither $C_{i}$ nor $C_{j}$ is decodable if and only if Equation (11) is violated for i=1,2 and Equation (12) is violated.

**Figure 2:**
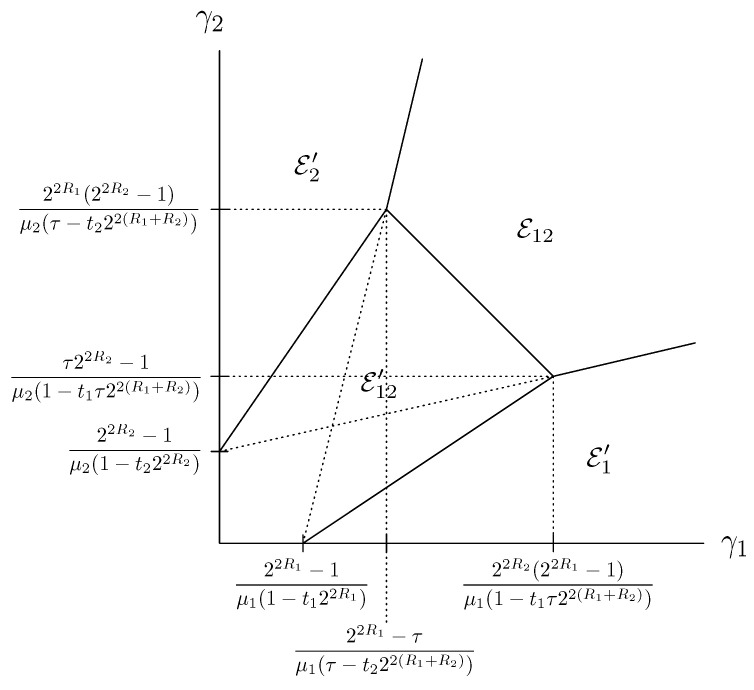
$\left(\gamma_{1},\gamma_{2}\right)$ pairs corresponding to outage events in HDA coding of uncorrelated Gaussian sources; $\tau =\frac{1-t_{2}}{1-t_{1}}$.

It can be verified that, when the sources are uncorrelated, Equations (11)–(14) describe regions of $\left(\gamma_{1},\gamma_{2}\right)$ for these decoding events as shown in Figure 2. For correlated sources, these regions not only depend on $\gamma_{1}$ and $\gamma_{2}$, but also on $\gamma_{12}$. We now proceed to evaluate MMSE under each event.

### 4.1. No Outage Event ($E_{12}$)

In this case, the digital channel codewords $C_{i}$, i=1,2 are correctly decoded at the receiver. Therefore $h_{1}{\tilde{X}}_{1}+h_{2}{\tilde{X}}_{2}$ can be perfectly canceled from the received signal Y to obtain the residual $\tilde{Y}=\left({\tilde{Y}}_{1},\cdots ,{\tilde{Y}}_{n}\right)$, where

$${\tilde{Y}}_{k}=h_{1}{\tilde{Z}}_{1,k}+h_{2}{\tilde{Z}}_{2,k}+W_{k},\;k=1,\cdots ,n,$$

and the source components $\left(S_{1},S_{2}\right)$ can be estimated from the recovered source codewords ${\tilde{S}}_{1}$ and ${\tilde{S}}_{2}$, and the residual sequence $\tilde{Y}$. The asymptotically optimal estimator is linear, and therefore the estimated source sequence $S_{i}$ is given by

$$\begin{array}{cc} {\hat{S}}_{i,k} & =q_{i1}{\tilde{S}}_{1,k}+q_{i2}{\tilde{S}}_{2,k}+q_{i3}{\tilde{Y}}_{k}\;k=1,\cdots ,n, \end{array}$$

where $q_{i,1}$, $q_{i,2}$, and $q_{i,3}$ are the coefficients of the optimal linear estimator. The MSE of the optimal estimator is

(15) $$\begin{array}{c} d_{i}\left(E_{12}|R_{1},R_{2},t_{1},t_{2}\right)=\sigma^{2}-q_{i1}c_{1}-q_{i2}c_{2}-q_{i3}c_{3},\;i=1,2, \end{array}$$

where $q_{il}$ and $c_{l}$, l=1,2,3 are found in Appendix C.1. Now, for given ($R_{1},R_{2},t_{1},t_{2})$, the total FA-MMSE can be found by evaluating

(16) $${\bar{d}}_{i}\left(E_{12}|R_{1},R_{2},t_{1},t_{2}\right)=\frac{1}{2}\int_{E_{12}}\sum_{i=1}^{2}d_{i}\left(E_{12}|R_{1},R_{2},t_{1},t_{2}\right)p_{1}\left(h_{1}\right)p_{2}\left(h_{2}\right)dh_{1}dh_{2}.$$

**Remark** **1.**

*1.*  *For the special case of uncoded transmission, we can set $R_{1}=R_{2}=0$ and $t_{1}=t_{2}=1$ ($\rho_{z}=0$).*
*2.*  *On the other hand, by setting $t_{1}=t_{2}=0$, we obtain the achievable MMSE of a purely digital SSC coding system. However, for correlated sources, this MMSE is less than that given by the Lemma 1. This is because the digital encoder in Figure 1 does not achieve a distributed coding gain, as does the digital encoder in Section 3. To achieve a coding gain, a distributed VQ must be used, but this will render the quantization errors of the two source uncorrelated, preventing us from exploiting the correlation between the GMAC inputs to our advantage. The advantage of the HDA scheme in Figure 1 is its robustness against unknown CSI. In particular, when the two sources are correlated, so will be their quantization errors. This correlation allows for a form of statistical cooperation at the GMAC output as reflected by the appearance of $\rho_{z}$ in Equation (16), see Appendix C.*

### 4.2. Partial Outage Event ($E_{i}^{\prime }$)

Suppose only $C_{i}$ is decodable ($C_{j}$ is undecodable), i∈{1,2}. Upon decoding $C_{i}$, the decoder computes $\tilde{Y}=Y-h_{i}\tilde{X_{i}}$ to obtain the residuals

(17) $${\tilde{Y}}_{k}=h_{i}U_{i,k}+h_{j}\left(U_{j,k}+{\tilde{X}}_{j,k}\right)+W_{k},\;k=1,\cdots ,n.$$

The optimal estimates of the source sequences are

$$\begin{array}{cc} {\hat{S}}_{i,k} & =q_{i1}{\tilde{S}}_{i,k}+q_{i2}{\tilde{Y}}_{k},\\ {\hat{S}}_{j,k} & =q_{i1}^{\prime }{\tilde{S}}_{i,k}+q_{i2}^{\prime }{\tilde{Y}}_{k}. \end{array}$$

and the corresponding MSEs are

$$\begin{array}{cc} d_{i}\left(E_{i}^{\prime }|R_{1},R2,t_{1},t_{2}\right) & =\sigma^{2}-q_{i1}c_{1}-q_{i2}c_{2},\\ d_{j}\left(E_{i}^{\prime }|R_{1},R2,t_{1},t_{2}\right) & =\sigma^{2}-q_{i1}^{\prime }c_{1}^{\prime }-q_{i2}^{\prime }c_{2}^{\prime }, \end{array}$$

where $q_{il}$, $q_{il}^{\prime }$, $c_{l}$ and $c_{l}^{\prime }$, l=1,2 are found in Appendix C.2. The total FA-MMSE can be found by evaluating

(18) $$\bar{d}\left(E_{i}^{\prime }|R_{1},R_{2},t_{1},t_{2}\right)=\frac{1}{2}\sum_{i=1}^{2}\int_{E_{i}^{\prime }}\left[d_{i}\left(E_{i}^{\prime }|R_{1},R_{2},t_{1},t_{2}\right)+d_{j}\left(E_{i}^{\prime }|R_{1},R_{2},t_{1},t_{2}\right)\right]p_{1}\left(h_{1}\right)p_{2}\left(h_{2}\right)dh_{1}dh_{2}.$$

### 4.3. Total Outage Event ($E_{12}^{\prime }$)

In this case, neither digital codeword is decodable, and the source sequences are reconstructed as

$$\begin{array}{c} {\hat{S}}_{i,k}=q_{i}Y_{k}\;k=1,\cdots ,n, \end{array}$$

i=1,2. The MSE of the optimal estimator is

$$\begin{array}{c} d_{i}\left(E_{12}^{\prime }|R_{1},R_{2},t_{1},t_{2}\right)=\sigma^{2}-q_{i}c_{i}^{*} \end{array}$$

where $q_{i}$ and $c_{i}$ are found in Appendix C.3. For given $\left(R_{1},R_{2},t_{1},t_{2}\right)$, the total FA-MMSE can be found by evaluating

$$\begin{array}{c} \bar{d}\left(E_{12}^{\prime }|R_{1},R_{2},t_{1},t_{2}\right)=\frac{1}{2}\sum_{i}\int_{E^{\prime }12}d_{i}\left(E_{12}^{\prime }|R_{1},R_{2},t_{1},t_{2}\right)p_{1}\left(h_{1}\right)p_{2}\left(h_{2}\right)dh_{1}dh_{2}. \end{array}$$

Finally, the total FA-MMSE of the superposition-based HDA scheme can be obtained by solving

(19) $$\begin{array}{c} D^{HDA(sup)}\left(P_{1},P_{2}\right)=\min_{R_{1},R_{2},t_{1},t_{2}}\bar{d}\left(R_{1},R_{2},t_{1},t_{2}\right), \end{array}$$

where

$$\begin{array}{c} \bar{d}\left(R_{1},R_{2},t_{1},t_{2}\right)=\bar{d}\left(E_{12}|R_{1},R_{2},t_{1},t_{2}\right)+\bar{d}\left(E_{1}^{\prime }|R_{1},R_{2},t_{1},t_{2}\right)+\bar{d}\left(E_{2}^{\prime }|R_{1},R_{2},t_{1},t_{2}\right)+\bar{d}\left(E_{12}^{\prime }|R_{1},R_{2},t_{1},t_{2}\right). \end{array}$$

## 5. JSC Coding

### 5.1. JSC-VQ

**Figure 3:**
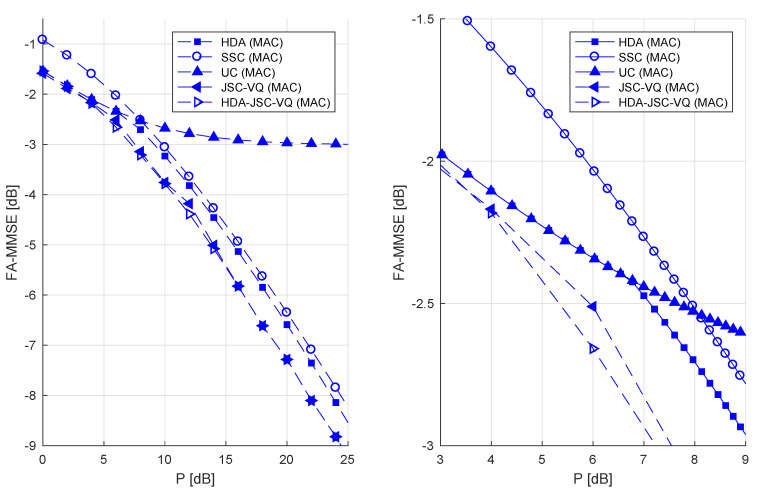
Fading-averaged (FA)-mean minimum square error (MMSE) for uncorrelated unit-variance Gaussian sources and Rayleigh-fading GMAC with $\bar{\Gamma }=1.0$.

**Figure 4:**
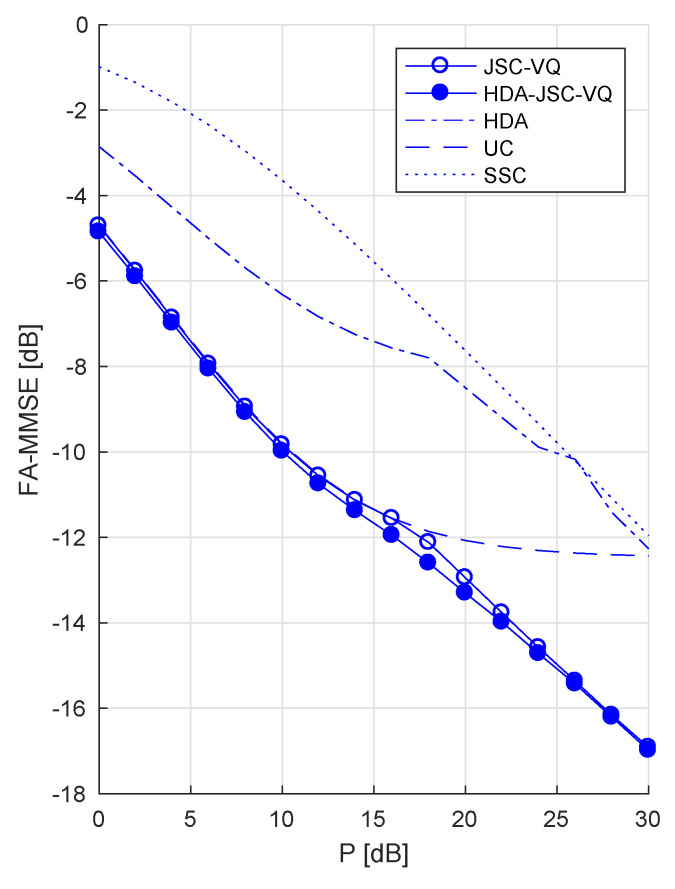
FA-MMSE for correlated unit-variance Gaussian sources with ρ=0.9 and Rayleigh-fading GMAC with $\bar{\Gamma }=1.0$.

One problem of digital coding with no knowledge of CSI at the transmitter is the unavoidable outage condition, which also exists in HDA coding schemes that use the quantization error of the digital encoder as the analog component. Although the problem does not exist in uncoded transmission, on a GMAC, uncoded transmission becomes inferior to SSC coding as the CSNR increases (for example, see Figure 3 and Figure 4). A simple way to improve the performance of uncoded transmission at high CSNR is suggested in [10]. Rate-distortion optimal VQ is applied to each source to be transmitted over the GMAC; the VQ codewords are scaled to meet the individual power constraints and directly transmitted over the channel. As the source codewords and channel codewords are the same in this case, optimal detection at the receiver can be used for joint source-channel decoding. More importantly, even if detection of VQ codewords fails, some estimate of the sources can still obtained from the observed channel output. The interest in [10] is the transmission of correlated sources over a NF-GMAC, or equivalently a BF-GMAC with CSI available at the transmitters. However, as we will demonstrate here, this approach can outperform HDA coding, even when the sources are uncorrelated, i.e., when the transmitters do not observe instantaneous CSI. In the following, we determine the FA-MMSE for this joint source-channel VQ (JSC-VQ) scheme. Our analysis considers the general case of two correlated Gaussian sources with the correlation coefficient ρ (the result for uncorrelated source can be obtained by setting ρ=0.) The JSC-VQ scheme has an additional advantage with correlated sources, since it allows the two correlated sources to statistically cooperate over a GMAC, i.e., create on the average constructive interference at the channel output. Furthermore, whenever one of the codewords is decoded correctly, the effective CSNR for the other codeword increases. As in HDA coding, the achievable MMSE is determined by three possible outage conditions at the decoder. However, the advantage here is that, even when none of the two codewords can be decoded correctly some estimates of the two sources can still be obtained from the observed channel output.

The JSC-VQ encoder *i* vector-quantizes the source sequence $S_{i}$ using a rate-$R_{i}$ codebook, scales the resulting codeword $U_{i}^{o}$ to satisfy its power constraint $P_{i}$, and transmits the scaled codeword $X_{i}=\alpha_{i}U_{i}^{o}$ over the BF-GMAC, where

(20) $$\alpha_{i}=\sqrt{\frac{P_{i}}{\sigma^{2}\left(1-2^{-2R_{i}}\right)}},$$

i=1,2. Note that, when $S_{1}$ and $S_{2}$ are correlated, so will be the channel inputs $X_{1}$ and $X_{2}$, and therefore the advantage of this scheme. Upon observing the resulting channel output Y, the decoder first uses the same VQ codebooks used by the encoders to jointly detect the transmitted codewords $\left(U_{1}^{o},U_{2}^{o}\right)$ by considering their correlation (detection step). In the second step, the detected VQ codewords are used to estimate the source sequences $S_{1}$ and $S_{2}$ (estimation step). Note that ($S_{1},S_{2},U_{1}^{o},U_{2}^{o},Y$) are asymptotically jointly Gaussian, and therefore the optimal (MMSE) estimator is linear. Let the codeword pair found in the detection step be $\left({\hat{U}}_{1},{\hat{U}}_{2}\right)$. In general, the estimated source sequences are given by

(21) $$\begin{array}{c} {\hat{S}}_{i}=q_{i1}{\hat{U}}_{1}+q_{i2}{\hat{U}}_{2}+q_{i3}Y,\;i=1,2. \end{array}$$

where coefficients $q_{i1}$, $q_{i2}$, and $q_{i3}$ of the optimal linear estimator are to be determined. For given $\left(R_{1},R_{2},\alpha_{1},\alpha_{2}\right)$ used by the encoders, it is not guaranteed that $\left(U_{1}^{o},U_{2}^{o}\right)$ can be correctly decoded in all channel states, and therefore $q_{i1}$, $q_{i2}$, and $q_{i3}$ will depend on the state (outage event) of the decoder. Let $H_{12}$, $H_{i}^{\prime }$, and $H_{12}^{\prime }$, respectively, be the set of $\left(h_{1},h_{2}\right)$, for which the outage events $E_{12}$, $E_{i}^{\prime }$, and $E_{12}^{\prime }$ occur for given (fixed) $\left(R_{1},R_{2},\alpha_{1},\alpha_{2}\right)$. Define the encoder parameter vector $t=(R_{1},R_{2},\alpha_{1},\alpha_{2})$. The FA-MMSE for given t can then be given by

(22) $$\begin{array}{cc} \bar{d}\left(t\right) & =\frac{1}{2}\sum_{i=1}^{2}\left\{\int_{H_{12}}d_{i}\left(E_{12}|t\right)dh+\int_{H_{1}^{\prime }}d_{i}\left(E_{1}^{\prime }|t\right)dh+\int_{H_{2}^{\prime }}d_{i}\left(E_{2}^{\prime }|t\right)dh+\int_{H_{12}^{\prime }}d_{i}\left(E_{12}^{\prime }|t\right)dh\right\}. \end{array}$$

The minimum achievable FA-MMSE can be found by

(23) $$\begin{array}{c} D^{JSC-VQ}\left(P_{1},P_{2}\right)=\min_{t}\bar{d}\left(t\right). \end{array}$$

We next consider all possible outage events to determine the conditional MMSEs in Equation (22).

- No-outage event $E_{12}$: The set of all rate pairs for which $E_{12}$ occurs are given by the following lemma.
  **Lemma** **2.** *For given $\left(P_{1},P_{2}\right)$, $\left(h_{1},h_{2}\right)$, $\left(\alpha_{1},\alpha_{2}\right)$, and ρ, both of the source-channel VQ codewords can be detected with an asymptotically vanishing error probability, if $\left(R_{1},R_{2}\right)$ satisfy*
  (24) $$\begin{array}{cc} R_{i} & <\frac{1}{2}\log_{2}\left(\frac{\left(1-{\tilde{\rho }}^{2}\right)P_{i}\Gamma_{i}+1}{1-{\tilde{\rho }}^{2}}\right)\;i=1,2\\ R_{1}+R_{2} & <\frac{1}{2}\log_{2}\left(\frac{P_{2}\Gamma_{1}+P_{2}\Gamma_{2}+2\tilde{\rho }\Gamma_{12}\sqrt{P_{1}P_{2}}+1}{1-{\tilde{\rho }}^{2}}\right), \end{array}$$
  *where $\tilde{\rho }$ is given by Equation (9).*
  **Proof** **.** See Appendix D. □
  Denote all $\left(R_{1},R_{2}\right)$ pairs that satisfy the above constraints by $R(E_{12})$. Let the codeword pair found in the detection step be $\left({\hat{U}}_{1},{\hat{U}}_{2}\right)$. If $\left(R_{1},R_{2}\right)\in R\left(E_{12}\right)$ then ${\hat{U}}_{1}=U_{1}^{o}$ and ${\hat{U}}_{2}=U_{2}^{o}$. In this case, the linear estimator need not use the channel output Y ($q_{i3}=0$), and the source sequences can be reconstructed as
  $$\begin{array}{c} {\hat{S}}_{i}=q_{i1}U_{1}^{o}+q_{i2}U_{2}^{o},\;i=1,2 \end{array}$$
  The MMSE of the linear estimator for $\left(R_{1},R_{2}\right)\in R\left(E_{12}\right)$ is given by Equation (A24).
- *Partial outage event $E_{i}^{\prime }$*: The set of all rate pairs for which $E_{i}^{\prime }$ (i=1,2) occurs are given by the following lemma.
  **Theorem** **1.** *For given $\left(P_{1},P_{2}\right)$, $\left(h_{1},h_{2}\right)$, $\left(\alpha_{1},\alpha_{2}\right)$, and ρ, the codeword $U_{i}^{o}$ is decodable and $U_{j}^{o}$ is undecodable if and only if*
  (25) $$\begin{array}{cc} R_{i} & <\frac{1}{2}\log_{2}\left(\frac{P_{1}\Gamma_{1}+P_{2}\Gamma_{2}+2\Gamma_{12}\tilde{\rho }\sqrt{P_{1}P_{2}}+1}{\Gamma_{j}P_{j}\left(1-{\tilde{\rho }}^{2}\right)+1}\right) \end{array}$$
  (26) $$\begin{array}{cc} R_{j} & >\frac{1}{2}\log_{2}\left(\frac{\left(1-{\tilde{\rho }}^{2}\right)P_{j}\Gamma_{j}+1}{1-{\tilde{\rho }}^{2}}\right) \end{array}$$
  *for i∈{1,2}.*
  **Proof** **.** The proof, considering the case i=1 and j=2, is given in Appendix D. □
  Denote all $\left(R_{1},R_{2}\right)$ pairs which satisfy above constraints by $R(E_{i}^{\prime })$. If $\left(R_{1},R_{2}\right)\in R\left(E_{i}^{\prime }\right)$, then ${\hat{U}}_{i}=U_{i}^{o}$ and ${\hat{U}}_{i}=0$. The source sequences are reconstructed as
  $$\begin{array}{c} {\hat{S}}_{i}=q_{i1}U_{i}^{o}+q_{i3}Y,\;i=1,2. \end{array}$$
  The expressions for the MMSE of the linear estimator for $\left(R_{1},R_{2}\right)\in R\left(E_{i}^{\prime }\right)$ are given by Equations (A25) and (A26).
- Total outage event $E_{12}^{\prime }$: The set of all rate pairs for which $E_{12}^{\prime }$ occurs is given by the following lemma.
  **Lemma** **3.** *For given $\left(P_{1},P_{2}\right)$, $\left(h_{1},h_{2}\right)$, $\left(\alpha_{1},\alpha_{2}\right)$, and ρ, neither codeword can be decoded if*
  $$\begin{array}{cc} R_{1} & >\frac{1}{2}\log_{2}\left(\frac{\Gamma_{1}P_{1}+\Gamma_{2}P_{2}+2\Gamma_{12}\sqrt{P_{1}P_{2}})+1}{\Gamma_{2}P_{2}\left(1-{\tilde{\rho }}^{2}\right)+1}\right)\\ R_{2} & >\frac{1}{2}\log_{2}\left(\frac{\Gamma_{2}P_{1}+\Gamma_{2}P_{2}+2\Gamma_{12}\sqrt{P_{1}P_{2}})+1}{\Gamma_{1}P_{1}\left(1-{\tilde{\rho }}^{2}\right)+1}\right)\\ R_{1}+R_{2} & >\frac{1}{2}\log_{2}\left(\frac{\Gamma_{1}P_{1}+\Gamma_{2}P_{2}+2\Gamma_{12}\tilde{\rho }\sqrt{P_{1}P_{2}})+1}{\left(1-{\tilde{\rho }}^{2}\right)}\right). \end{array}$$
  **Proof** **.** Follows from Equations (24) and (25) in the previous two lemmas. □
  Denote all $\left(R_{1},R_{2}\right)$ pairs which satisfy above constraints by $R(E_{12}^{\prime })$. The source sequences are reconstructed as
  $$\begin{array}{c} {\hat{S}}_{i}=q_{i3}Y,\;i=1,2. \end{array}$$
  The expressions for the MMSE of the linear estimator for $\left(R_{1},R_{2}\right)\in R\left(E_{12}^{\prime }\right)$ are given Equation (A27).

### 5.2. HDA-JSC-VQ Coding

Finally, we consider an HDA scheme based on the aforementioned JSC-VQ scheme, which possibly provides the lowest known upper bound to the distortion power function $D(P_{1},P_{2})$ for a pair of correlated Gaussian sources and a fading GMAC. In this scheme, a scaled (analog) version of each source is superimposed on the JSC-VQ codewords in the scheme discussed in Section 5.1. In particular, the resulting HDA scheme (which we will refer to as HDA-JSC-VQ coding) can be shown to outperform the JSC-VQ at all CSNRs on a non-fading GMAC [10]. This improvement can be attributed to the optimality of analog transmission as SNR →0. In this section, we determine the minimum achievable FA-MMSE of the HDA-JSC-VQ coding over the BF-GMAC. In this case, there is an additional gain due to combining an analog transmission with JSV-VQ as this prevents the complete outages that would otherwise occur with JSV-VQ. We will also demonstrate that this scheme achieves a better FA-MMSE than any other known scheme, even for the uncorrelated Gaussian sources.

Using the same notation as in the previous section, the channel codeword generated by the encoder *i* of our HDA-JSC-VQ system can be given by

$$\begin{array}{c} X_{i}=\alpha_{i}S_{i}+\beta_{i}U_{i}^{o}, \end{array}$$

where $U_{i}^{o}$ is the VQ codeword for $S_{i}$, and $\alpha_{i}$ and $\beta_{i}$ are constants. Using the transmit power constraint, we have

$$\begin{array}{c} \beta_{i}=\sqrt{\frac{P_{i}-\alpha_{i}^{2}\sigma^{2}2^{-2R_{i}}}{\sigma^{2}\left(1-2^{-2R_{i}}\right)}}-\alpha_{i}, \end{array}$$

and the digital–analog power allocation factor $\alpha_{i}$ must be chosen to minimize the FA-MMSE. Given the observed channel output $Y=h_{1}X_{1}+h_{2}X_{2}+W$, the receiver first decodes $\left(U_{1}^{o},U_{2}^{o}\right)$ as $\left({\hat{U}}_{1},{\hat{U}}_{2}\right)$ and then linearly estimates the source sequence $S_{i}$ as

$$\begin{array}{c} {\hat{S}}_{i}=q_{i1}{\hat{U}}_{1}+q_{i2}{\hat{U}}_{2}+q_{i3}Y, \end{array}$$

where the coefficients $q_{il}$, l=1,2 are chosen to minimize the reconstruction MMSE for given $\left(h_{1},h_{2}\right)$. The evaluation FA-MMSE can proceed as in the case of JSC-VQ system. Specifically, by redefinition of the encoder parameters as $t=(R_{1},R_{2},\alpha_{1},\beta_{1},\alpha_{2},\beta_{2})$, the conditional FA-MMSE can be found by Equation (22). The minimum achievable FA-MMSE in this case is given by

(27) $$\begin{array}{c} D^{HDA-JSC-VQ}\left(P_{1},P_{2}\right)=\min_{t}\bar{d}\left(t\right). \end{array}$$

What remains is to determine the conditional MMSEs in Equation (22), by considering every possible outage event. We obtain the required MMSEs by proving a set of lemmas.

- *No-outage event $E_{12}$*: For fixed $\left(h_{1},h_{2}\right)$, the bounds on VQ rates required to guarantee error-free decoding of $\left(U_{1}^{o},U_{2}^{o}\right)$ can be found through a slight generalization of the results in ([10], Theorem IV.6) to account for channel-gains and complex-valued random variables. In particular we can prove the following lemma.
  **Lemma** **4.** *For given $\left(P_{1},P_{2}\right)$, $\left(\alpha_{1},\beta_{1}\right)$, $\left(\alpha_{2},\beta_{2}\right)$, $\left(h_{1},h_{2}\right)$, ρ, the VQ codeword-pair $\left(U_{1}^{o},U_{2}^{o}\right)$ can be decoded error-free whenever $\left(R_{1},R_{2}\right)$ satisfies*
  $$\begin{array}{cc} R_{1} & <\frac{1}{2}\log_{2}\left(\frac{|\beta_{1}^{\prime }{|}^{2}k_{11}\left(1-{\tilde{\rho }}^{2}\right)+N^{\prime }}{N^{\prime }\left(1-{\tilde{\rho }}^{2}\right)}\right)\\ R_{2} & <\frac{1}{2}\log_{2}\left(\frac{|\beta_{2}^{\prime }{|}^{2}k_{22}\left(1-{\tilde{\rho }}^{2}\right)+N^{\prime }}{N^{\prime }\left(1-{\tilde{\rho }}^{2}\right)}\right)\\ R_{1}+R_{2} & <\frac{1}{2}\log_{2}\left(\frac{|\beta_{1}^{\prime }{|}^{2}k_{11}+|\beta_{2}^{\prime }{|}^{2}k_{22}+2Re\left\{\beta_{1}^{\prime }{\left(\beta_{2}^{\prime }\right)}^{*}\right\}\tilde{\rho }\sqrt{k_{11}k_{22}})+N^{\prime }}{N^{\prime }\left(1-{\tilde{\rho }}^{2}\right)}\right), \end{array}$$
  *where*
  $$\begin{array}{c} N^{\prime }=|h_{1}{|}^{2}\alpha_{1}^{2}\nu_{1}+{\left|h_{2}\right|}^{2}\alpha_{2}^{2}\nu_{2}+2Re\left\{h_{1}^{*}h_{2}\right\}\alpha_{1}\alpha_{2}\nu_{3}+N, \end{array}$$
  *$\nu_{1}$, $\nu_{1}$, and $\nu_{3}$ are given by the set of expression following (45) in [10], but with*
  (28) $$\begin{array}{cc} \beta_{1}^{\prime } & =h_{1}\alpha_{1}\left(1-a_{1}\tilde{\rho }\right)+h_{1}\beta_{1}+h_{2}\alpha_{2}a_{2} \end{array}$$
  (29) $$\begin{array}{cc} \beta_{2}^{\prime } & =h_{2}\alpha_{2}\left(1-a_{2}\tilde{\rho }\right)+h_{2}\beta_{2}+h_{1}\alpha_{1}a_{1}, \end{array}$$
  *for $a_{1}$ and $a_{2}$ given by Equations (48)–(50) in [10].*
  For $\left(R_{1},R_{2}\right)$ satisfying the bounds in Lemma 4, the minimum achievable MMSE is given by Equation (A28).
- Partial outage event $E_{i}^{\prime }$: Rate pairs $\left(R_{1},R_{2}\right)$ for which only one codeword can be decoded is given by the following lemma.
  **Lemma** **5.** *For given $\left(P_{1},P_{2}\right)$, $\left(\alpha_{1},\beta_{1}\right)$, $\left(\alpha_{2},\beta_{2}\right)$, $\left(h_{1},h_{2}\right)$, and ρ, the necessary and sufficient conditions where only codeword $U_{i}^{o}$, but not $U_{j}^{o}$, is correctly decodable are*
  $$\begin{array}{cc} R_{i} & <\frac{1}{2}\log_{2}\left(\frac{|\beta_{1}^{\prime }{|}^{2}k_{11}+|\beta_{2}^{\prime }{|}^{2}k_{22}+2Re\left\{\beta_{1}^{\prime }\left(\beta_{2}^{\prime *}\right)\right\}\tilde{\rho }\sqrt{k_{11}k_{22}})+N^{\prime }}{|\beta_{i}^{\prime }{|}^{2}k_{jj}\left(1-{\tilde{\rho }}^{2}\right)+N^{\prime }}\right)\\ R_{j} & >\frac{1}{2}\log_{2}\left(\frac{|\beta_{i^{\prime }}^{\prime }{|}^{2}k_{jj}\left(1-{\tilde{\rho }}^{2}\right)+N^{\prime }}{N^{\prime }\left(1-{\tilde{\rho }}^{2}\right)}\right) \end{array}$$
  For $\left(R_{1},R_{2}\right)$ satisfying the bounds in Lemma 5, the minimum achievable MMSE is given by Equation (A29).
- Total outage event $E_{12}^{\prime }$: Rate pairs $\left(R_{1},R_{2}\right)$ for which neither codeword can be decoded is given by the following lemma.
  **Lemma** **6.** *For given $\left(P_{1},P_{2}\right)$, $\left(\alpha_{1},\beta_{1}\right)$, $\left(\alpha_{2},\beta_{2}\right)$, $\left(h_{1},h_{2}\right)$, and ρ, neither $U_{i}^{o}$ nor $U_{j}^{o}$ can be decoded if*
  $$\begin{array}{cc} R_{1} & >\frac{1}{2}\log_{2}\left(\frac{|\beta_{1}^{\prime }{|}^{2}k_{11}+|\beta_{2}^{\prime }{|}^{2}k_{22}+2Re\left\{\beta_{1}^{\prime }{\left(\beta_{2}^{\prime }\right)}^{*}\right\}\tilde{\rho }\sqrt{k_{11}k_{22}})+N^{\prime }}{|\beta_{2}^{\prime }{|}^{2}k_{22}\left(1-{\tilde{\rho }}^{2}\right)+N^{\prime }}\right)\\ R_{2} & >\frac{1}{2}\log_{2}\left(\frac{|\beta_{1}^{\prime }{|}^{2}k_{11}+|\beta_{2}^{\prime }{|}^{2}k_{22}+2Re\left\{\beta_{1}^{\prime }{\left(\beta_{2}^{\prime }\right)}^{*}\right\}\tilde{\rho }\sqrt{k_{11}k_{22}})+N^{\prime }}{|\beta_{1}^{\prime }{|}^{2}k_{11}\left(1-{\tilde{\rho }}^{2}\right)+N^{\prime }}\right)\\ R_{1}+R_{2} & >\frac{1}{2}\log_{2}\left(\frac{|\beta_{1}^{\prime }{|}^{2}k_{11}+|\beta_{2}^{\prime }{|}^{2}k_{22}+2Re\left\{\beta_{1}^{\prime }{\left(\beta_{2}^{\prime }\right)}^{*}\right\}\tilde{\rho }\sqrt{k_{11}k_{22}})+N^{\prime }}{N^{\prime }\left(1-{\tilde{\rho }}^{2}\right)}\right). \end{array}$$
  For $\left(R_{1},R_{2}\right)$ satisfying the bounds in Lemma 6, the minimum achievable MMSE is given by Equation (A30).

Lemmas 4–6 can be proven (details omitted for brevity) by considering the “genie-aided” decoder argument in ([10], Appendix F), in conjunction with the rate conditions for three-types decoding events established in Appendix D of this paper. In particular, the decoding events of HDA-JSC-VQ scheme can be mapped to those of the JSC-VQ scheme in Section 5.1, by re-expressing the channel output in the form $Y=\beta_{1}^{\prime }U_{1}^{o}+\beta_{2}^{\prime }U_{2}^{o}+W^{\prime }$ such that the additive noise $W^{\prime }$ satisfies the properties required by the proofs in Appendix D. It can be verified (see [10], Lemma F.1) that the desired representation for Y is obtained by choosing $\beta_{1}^{\prime }$ and $\beta_{2}^{\prime }$ as in Equations (28) and (29), respectively.

## 6. Comparison of Bounds and Discussion

**Figure 5:**
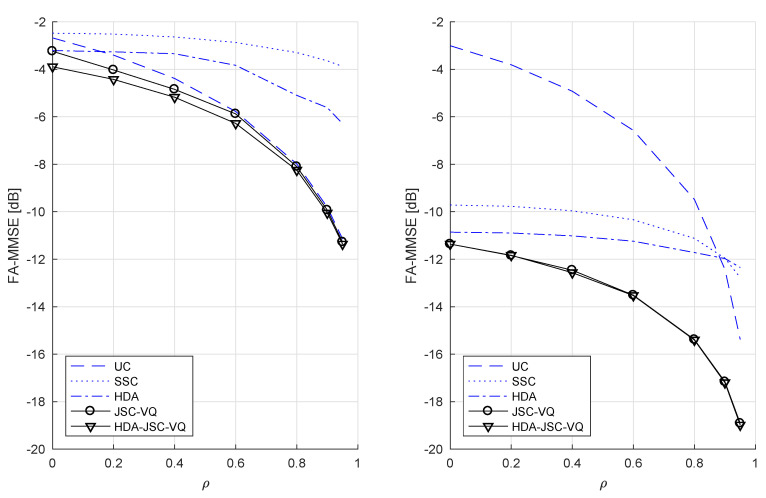
P=10 dB (**left**) and P=30 dB (**right**).

In summary, Equations (3), (19), (23), and (27) are all computable upper bounds to the (unknown) distortion power function $D(P_{1},P_{2})$ of Gaussian sources and a Rayleigh fading GMAC, under the constraint that CSI is not observable at the transmitters (the function minimizations required to evaluate these bounds have been carried out by using global optimization software). These bounds have been numerically evaluated for certain examples and the results are presented in Figure 3, Figure 4 and Figure 5. For simplicity of presentation, we consider the symmetric case of $P_{1}=P_{2}$ and ${\bar{\Gamma }}_{1}={\bar{\Gamma }}_{2}=1$ (CSNR is thus the same as *P*). We consider sources with variance $\sigma^{2}=1$.

Recall that if CSI is available at the transmitters as well, SSC coding is optimal for uncorrelated sources, whereas uncoded transmission is not. The performance curves in Figure 3 confirm that this is not the case if CSI is not available to the transmitters. The lack of CSI forces the coded transmitters to choose an encoding rate (based on prior knowledge of the CSI distribution) to minimize the MSE considering the unavoidable receiver outages. In the lower-power regime where outage probability is very high, uncoded transmission therefore achieves a better distortion than coded transmission. At the high-power regime, however, the two sources cannot be completely separated from the sum created by the MAC if transmitted completely uncoded and hence the MMSE of uncoded system reaches a constant (which in this case is $\sigma^{2}/2=0.5$ or −3 dB.) As seen in Figure 3 (right) conventional HDA coding essentially remains uncoded transmission up to about P=6.5 dB (all power allocated to analog part), and then diverges thereafter due to the increasing power allocation to the digital part. While the exact DPF is not known, the HDA-JSC-VQ provides lowest known upper bound to the DPF. Note that JSC-VQ bound coincides with the HDA-JSC-VQ bound for high *P* where all available power gets allocated to the VQ codewords.

For correlated sources, SSC coding would not be optimal even if CSI was known to the transmitters. Figure 4 shows that at ρ=0.9, SSC coding has a significant gap (~3–6 dB) to the JSC-VQ and HDA-JSC-VQ bounds. Figure 5 left and right shows the achievable FA-MMMSE of each system as a function of the source correlation coefficient, at low and high transmitter powers, respectively. It is known that on a fixed GMAC, uncoded transmission is optimal for power-to-noise ratios $P/N\leq \frac{\rho }{1-\rho^{2}}$ [10]. For example, if ρ=0.9, uncoded transmission must be optimal for the fixed channel if P/N≤6.75 dB. Clearly, uncoded transmission can only be optimal for a fraction of time in a system with fading and fixed (non-adaptive) transmitters and therefore cannot be optimal in a FA-MMSE sense. As Figure 4 shows, the uncoded system performs identical to the JSC-VQ system in the low-power regime (*P* less than about 15 dB in this example). This should be expected, as the the limiting optimal JSC-VQ system (as P→0) is the uncoded system, i.e., the receiver operates in outage nearly all the time, and therefore the optimal system has a rate that approaches infinity. The HDA-JSC-VQ system, on the other hand, exhibits a different behavior. The analog–digital power allocation and VQ rates in the HDA-JSC-VQ system ensure that the receiver achieves an optimal operating point with respect to all four outage events. This allows the HDA-JSC-VQ system to achieve a lower FA-MMSE at a given *P*, compared to both JSC-VQ and uncoded system in the lower power regime, as evident from Figure 4.

**Figure 6:**
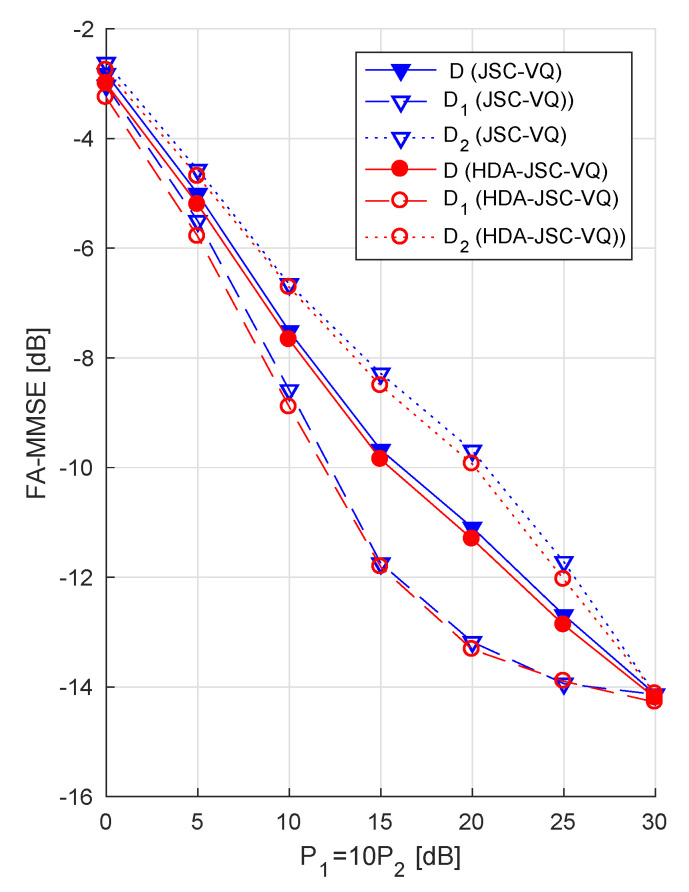
FA-MMSE for correlated unit-variance Gaussian sources with ρ=0.9 and $\bar{\Gamma }=1.0$, when the output power of the transmitter for source 2 has 10 dB lower than that for source 1 (i.e., $P_{1}=10P_{2}$).

So far, we have considered the symmetric case, that is, $P_{1}=P_{2}$. As an example for an asymmetric case, Figure 6 shows the FA-MMSE of JSC-VQ and HDA-JSC-VQ schemes for $P_{2}=P_{1}/10$ (i.e., $P_{2}$ is 10 dB below $P_{1}$) when ρ=0.9 and $\bar{\Gamma }=1$. Note that at high CSNR, the high correlation between the two sources allows the receiver to achieve $D_{2}\approx D_{1}$, despite the transmitter powers being very different. When comparing this figure to Figure 4, note that the total transmitter output power ($P_{1}+P_{2}$) is lower here ($1.1P_{1}$ compared to $2P_{1}$), which explains the higher total FA-MMSE compared to Figure 4.

Although practical code construction methods related to our problem have been reported in previous work, e.g., [18,19,20], those methods perform well only when CSI is available at both the transmitter and the receiver. Furthermore, [18,19] also suffer an additional performance loss due to being zero-delay coding schemes. The results presented in this paper serve as a guide to developing good practical multiple-access block codes for transmitting Gaussian-like sources in systems with no CSI at the transmitters. If the average CSNR is low, uncoded transmission can achieve nearly the same performance as an HDA-JSC-VQ system. The relative performance of the uncoded system improves with the source correlation. However, at moderate to high CSNRs, HDA-JSC-VQ will have a definite advantage, regardless of source correlation. Optimal VQ and typical sequence detection, as considered here to analyze HDA-JSC-VQ, are obviously not practically realizable. A potential approach to practically realizing a HDA-JSC-VQ system is by using trellis-coded quantization (TCQ) [21] at the transmitters (with optimal rates and power allocations found as described in this paper) and joint maximum likelihood (ML) sequence detection at the receiver [22]. On the one hand, TCQ allows computationally efficient way of quantizing long source sequences with distortion close to the distortion rate bound for optimal VQ; on the other hand, the joint detection of a pair of long VQ codewords can be efficiently implemented using a suitable variant of the Vitterbi algorithm operating on a combined trellises of two TCQs. Our preliminary experimental results suggest that this approach can achieve performance very close to the FA-MMSE bound derived in this paper. A complete set of experimental results will be reported in a future paper.

## Figures and Tables

**Figure A1:**
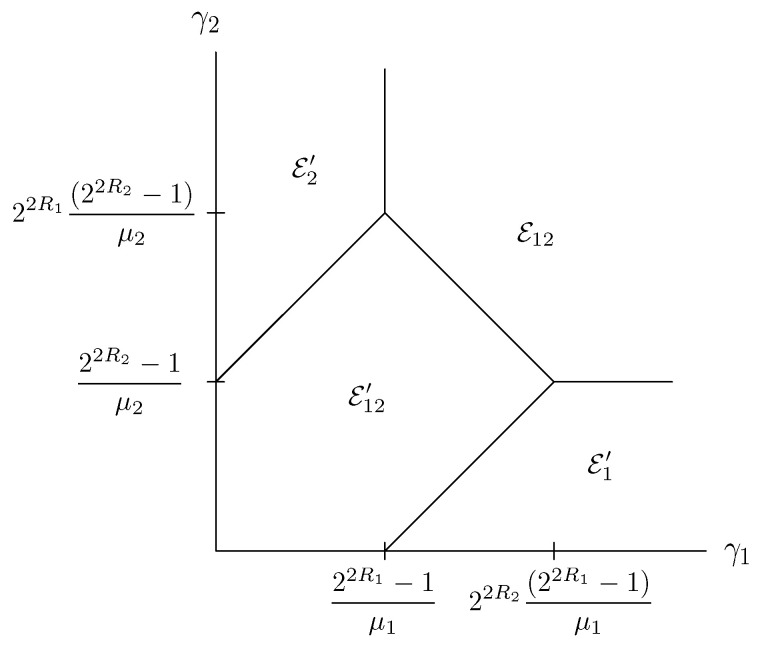
$\left(\gamma_{1},\gamma_{2}\right)$ pairs corresponding to outage events in separate source-channel (SSC) coding of Gaussian sources.

**Figure A2:**
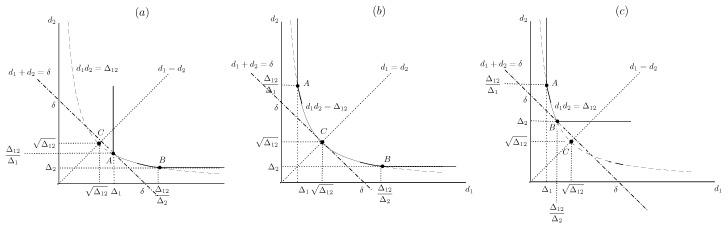
Distortion pairs achievable in distributed VQ of two correlated Gaussian sources at a fixed rate pair $\left(R_{1},R_{2}\right)$.

**Figure A3:**
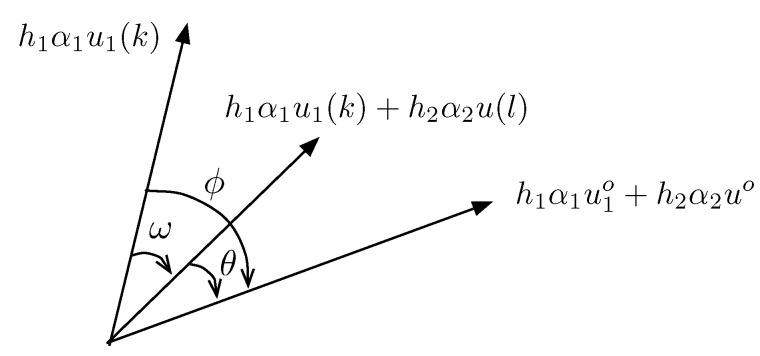
The definition of asymptotic angles used to prove Lemma A4.

## Appendix A. Outage Probabilities in SSC Coding of Gaussian Sources

From ([4], Equations 15.147–15.149), it directly follows that the the necessary and sufficient conditions for various outage events are as follows.

- $E_{12}$ (both codewords decodable):
  $$\begin{array}{cc} P_{i}\Gamma_{i} & \geq 2^{2R_{i}}-1,\;i=1,2\\ P_{1}\Gamma_{1}+P_{2}\Gamma_{2} & \geq 2^{2(R_{1}+R_{2})}-1 \end{array}$$
- $E_{i}^{\prime }$ (only one codeword decodable):
  $$\begin{array}{cc} \frac{P_{i}\Gamma_{i}}{1+P_{j}\Gamma_{j}} & \geq 2^{2R_{i}}-1,\;i=1,2\\ P_{j}\Gamma_{j} & <2^{2R_{j}}-1 \end{array}$$
- $E_{12}^{\prime }$ (neither codeword decodable):
  $$\begin{array}{cc} \frac{P_{i}\Gamma_{i}}{1+P_{j}\Gamma_{j}} & <2^{2R_{i}}-1,\;i=1,2\\ P_{1}\Gamma_{1}+P_{2}\Gamma_{2} & <2_{1}^{2(R_{1}+R_{2})}-1 \end{array}$$

It is straightforward to verify that $\left(\gamma_{1},\gamma_{2}\right)$ pairs corresponding to these events are as shown in Figure A1, where we define $\mu_{i}=P_{i}/N$.

The probability of the event E is given by

$$Pr\left(E|R_{1},R_{2}\right)=\int_{E}p_{1}\left(\gamma_{1}\right)p_{2}\left(\gamma_{2}\right)d\gamma_{1}d\gamma_{2}.$$

where, for Rayleigh fading, the pdf of $\gamma_{i}$ is $p_{i}\left(\gamma_{i}\right)=\frac{1}{\bar{\gamma }}\exp\left(-\frac{\gamma_{i}}{\bar{\gamma }}\right)$. By evaluating the integral over each region, both $Pr(E_{12}|R_{1},R_{2})$ and $Pr(E_{i}^{\prime }|R_{1},R_{2})$ can be obtained in closed-form. The former has cumbursome expression (not shown here), but the latter is given by

$$\begin{array}{cc} Pr(E_{i}^{\prime }|R_{1},R_{2}) & =e^{-\frac{2R_{i}\left(2^{2R_{j}}-1\right)}{\mu_{i}}}\left\{1-e^{-\frac{\left(2^{2R_{i}}-1\right)}{\mu_{j}}}\right\},\;i=1,2. \end{array}$$

Straightforwardly, $Pr\left(E_{12}^{\prime }|R_{1},R_{2}\right)=1-Pr\left(E_{12}|R_{1},R_{2}\right)-Pr\left(E_{1}^{\prime }|R_{1},R_{2}\right)-Pr\left(E_{2}^{\prime }|R_{1},R_{2}\right)$.

## Appendix B. Proof of Lemma 1

We first state a lemma that is required to prove Lemma 1.

**Lemma** **A1.**
*Let*

$$\Delta \left(x,y\right)=2^{-2x}\left(1-\rho^{2}+\rho^{2}2^{-2y}\right)$$

*and $R_{sum}=R_{1}+R_{2}$. Given a distributed VQ (for a pair of zero-mean, variance $\sigma^{2}$ Gaussian sources whose correlation coefficient is ρ) with rates $R_{1}$ and $R_{2}$, the distortion pair $\left(D_{1},D_{2}\right)$ is achievable if and only if*

(A1) $$\begin{array}{c} \left(D_{1},D_{2}\right)\in \left\{D_{1}\left(R_{1},R_{2}\right)\cap D_{2}\left(R_{1},R_{2}\right)\cap D_{\mathrm{prod}}\left(R_{1},R_{2}\right)\right\} \end{array}$$

*where*

$$\begin{array}{cc} D_{1}\left(R_{1},R_{2}\right) & =\left\{D_{1}:D_{1}\geq \sigma^{2}\Delta \left(R_{1},R_{2}\right)\right\}\\ D_{2}\left(R_{1},R_{2}\right) & =\left\{D_{2}:D_{2}\geq \sigma^{2}\Delta \left(R_{2},R_{1}\right)\right\}\\ D_{\mathrm{prod}}\left(R_{1},R_{2}\right) & =\left\{\left(D_{1},D_{2}\right):D_{1}D_{2}\geq \sigma^{4}\Delta \left(R_{sum},R_{sum}\right)\right\}. \end{array}$$

**Proof.** This lemma follows from the achievable rate region given by ([8], Theorem 1). □

Proof of Lemma 1: Let $\left(d_{1},d_{2}\right)$ be all distortion pairs achievable by a distributed VQ with rates $\left(R_{1},R_{2}\right)$. From Lemma A1, it follows that all achievable distortion pairs satisfy

(A2) $$\begin{array}{cc} d_{1} & \geq \Delta_{1}=\sigma^{2}\Delta \left(R_{1},R_{2}\right) \end{array}$$

(A3) $$\begin{array}{cc} d_{2} & \geq \Delta_{2}=\sigma^{2}\Delta \left(R_{2},R_{1}\right) \end{array}$$

(A4) $$\begin{array}{cc} d_{1}d_{2} & \geq \;\Delta_{12}=\sigma^{4}\Delta \left(R_{sum},R_{sum}\right). \end{array}$$

For a given $\left(R_{1},R_{2}\right)$ pair, all $\left(d_{1},d_{2}\right)$ that satisfy these constraints are above the curve shown in Figure A2 [achievable distortion region for $\left(R_{1},R_{2}\right)$]. The minimum achievable total MSE is found by minimizing $d_{1}+d_{2}$ subject to the constraints Equations (A2)–(A4) (feasibility set). It should be clear that the optimal solution occurs when δ is such that the line $d_{1}+d_{2}=\delta$ touches the boundary of the feasibility set. Depending on the relative values of $\Delta_{1}$, $\Delta_{2}$ and $\Delta_{12}$, this will occur at the point A, B, or C as follows,

*A*$\mathrm{if}\;\Delta_{2}<\sqrt{\Delta_{12}}<\Delta_{1}\;\mathrm{and}\;\delta =\Delta_{2}+\frac{\Delta_{12}}{\Delta_{2}}\;$ [Figure A2a],
*B*$\mathrm{if}\;\Delta_{1}<\sqrt{\Delta_{12}}<\Delta_{2}\;\mathrm{and}\;\delta =\Delta_{1}+\frac{\Delta_{12}}{\Delta_{1}}\;$ [Figure A2c],
*C*$\mathrm{if}\;\sqrt{\Delta_{12}}>\max\left\{\Delta_{1},\Delta_{2}\right\}\;\mathrm{and}\;\delta =2\sqrt{\Delta_{12}}\;$ [Figure A2b].

The inequalities in the first two statements are equivalent to $\max\left\{\Delta_{1},\Delta_{2}\right\}<\sqrt{\Delta_{12}}<\max\left\{\Delta_{1},\Delta_{2}\right\}$. Therefore, the minimum $d_{1}+d_{2}$ is

$$\delta^{*}=\Delta^{*}+\frac{\Delta_{12}}{\Delta^{*}},$$

where $\Delta^{*}$ as given by Equation (4).

## Appendix C. Optimal Linear Estimators for HDA Coding

In this appendix, we determine the MSE of the optimal linear estimator in conventional HDA coding.

1. No-outage event ($E_{12}$): Linear estimator used in the HDA system, estimates the source sample $S_{i,k}$, k=1,…,n using the observation vector $Y_{o}={\left({\tilde{S}}_{1,k}\;{\tilde{S}}_{2,k}\;\tilde{Y}\right)}^{T}$. Define the asymptotic autocovariance matrix $K={\bar{E\{Y_{o}Y_{o}^{H}\}}}_{n}$ and the cross-covariance vector $c_{i}={\bar{E\{{\tilde{S}}_{i,k}^{*}Y_{o}\}}}_{n}$. For optimal VQ of Gaussian sources, Equations (5)–(8) hold and the following can be verified.
  $$\begin{array}{cc} k_{11} & ={\bar{E\{|{\tilde{S}}_{1}{|}^{2}\}}}_{n}=\sigma^{2}\left(1-2^{-2R_{1}}\right)\\ k_{12} & =k_{21}={\bar{E\{{\tilde{S}}_{1}^{*}{\tilde{S}}_{2}\}}}_{n}=\sigma^{2}\rho \left(1-2^{-2R_{1}}\right)\left(1-2^{-2R_{2}}\right)\\ k_{13} & =k_{31}^{*}={\bar{E\{{\tilde{S}}_{1}^{*}\tilde{Y}\}}}_{n}=\alpha_{a2}h_{2}\rho 2^{-2R_{2}}k_{11}\\ k_{22} & ={\bar{E\{|{\tilde{S}}_{2}{|}^{2}\}}}_{n}=\sigma^{2}\left(1-2^{-2R_{2}}\right)\\ k_{23} & =k_{32}^{*}={\bar{E\{{\tilde{S}}_{2}^{*}\tilde{Y}\}}}_{n}=\alpha_{a1}h_{1}\rho 2^{-2R_{1}}k_{22}\\ k_{33} & ={\bar{E\{|\tilde{Y}{|}^{2}\}}}_{n}=\gamma_{1}t_{1}P_{1}+\gamma_{2}t_{2}P_{2}+2\gamma_{12}\rho_{z}\sqrt{t_{1}t_{2}P_{1}P_{2}}+N,\\ c_{11} & ={\bar{E\{S_{1}^{*}{\tilde{S}}_{1}\}}}_{n}=k_{11}\\ c_{12} & ={\bar{E\{S_{1}^{*}{\tilde{S}}_{2}\}}}_{n}=\rho k_{22}\\ c_{13} & =c_{31}^{*}={\bar{E\{S_{1}^{*}\tilde{Y}\}}}_{n}=\left(\alpha_{a1}h_{1}2^{-2R_{1}}+\alpha_{a2}h_{2}\rho 2^{-2R_{2}}\right)\sigma^{2}\\ c_{21} & ={\bar{E\{S_{2}^{*}{\tilde{S}}_{1}\}}}_{n}=\rho k_{11}\\ c_{22} & ={\bar{E\{S_{2}^{*}{\tilde{S}}_{2}\}}}_{n}=k_{22}\\ c_{23} & =c_{32}^{*}={\bar{E\{S_{2}^{*}\tilde{Y}\}}}_{n}=\left(\alpha_{a2}h_{2}2^{-2R_{2}}+\alpha_{a1}h_{1}\rho 2^{-2R_{1}}\right)\sigma^{2}. \end{array}$$
  Let the optimal linear estimator coefficients be $q_{i}={\left(q_{i1}\;q_{i2}\;q_{13}\right)}^{T}$. Then, we have $q_{i}=K^{-1}c_{i}^{*}$, i=1,2.
2. Partial outage event ($E_{i}^{\prime }$): In this case, define $Y_{o}={\left({\tilde{S}}_{i,k}\;{\tilde{Y}}_{k}\right)}^{T}$, i∈{1,2}, and $K_{i}={\bar{E\{Y_{o}Y_{o}^{H}\}}}_{n}$, which is a 2×2 matrix whose elements are
  $$\begin{array}{cc} k_{11} & ={\bar{E\{|{\tilde{S}}_{i}{|}^{2}\}}}_{n}=\sigma^{2}\left(1-2^{-2R_{i}}\right)\\ k_{12} & =k_{21}^{*}={\bar{E\{{\tilde{S}}_{i}^{*}\tilde{Y}\}}}_{n}=\alpha_{aj}h_{j}\rho 2^{-2R_{j}}k_{11}\\ k_{22} & ={\bar{E\{|\tilde{Y}{|}^{2}\}}}_{n}=\gamma_{i}t_{i}P_{i}+\gamma_{j}P_{j}+2\gamma_{12}\rho_{z}\sqrt{t_{1}t_{2}P_{1}P_{2}}+N. \end{array}$$
  Let optimal linear estimates for $S_{i,k}$ and $S_{j,k}$, k=1,…,n be $q_{i}={\left(q_{i1}\;q_{i2}\right)}^{T}$ and $q_{i}^{\prime }={\left(q_{i1}^{\prime }\;q_{i2}^{\prime }\right)}^{T}$. Then, we have $q_{i}=K_{i}^{-1}c_{i}^{*}$ and $q_{j}=K_{i}^{-1}{\left(c_{i}^{\prime }\right)}^{*}$, where $c_{i}={\left(c_{i1}\;c_{i2}\right)}^{T}$ and $c_{i}^{\prime }={\left(c_{i1}^{\prime }\;c_{i2}^{\prime }\right)}^{T}$ with
  $$\begin{array}{cc} c_{i1} & ={\bar{E\{S_{i}^{*}{\tilde{S}}_{i}\}}}_{n}=\rho k_{11}\\ c_{i2} & ={\bar{E\{S_{i}^{*}\tilde{Y}\}}}_{n}=\alpha_{ai}h_{i}\sigma^{2}2^{-2R_{i}}+\alpha_{aj}h_{j}\rho \sigma^{2}2^{-2R_{j}}\\ c_{i1}^{\prime } & ={\bar{E\{S_{j}^{*}{\tilde{S}}_{i}\}}}_{n}=\rho k_{11}\\ c_{i2}^{\prime } & ={\bar{E\{S_{j}^{*}\tilde{Y}\}}}_{n}=\alpha_{ai}h_{i}\rho \sigma^{2}2^{-2R_{i}}+\alpha_{aj}h_{j}\sigma^{2}2^{-2R_{j}}. \end{array}$$
3. *Total outage event ($E_{12}^{\prime }$)*: The optimal source estimates are given by ${\hat{S}}_{i,k}=q_{i}Y_{k}$, i=1,2 for k=1,…,n, where $q_{i}=k^{-1}c_{i}$ and
  $$\begin{array}{cc} k & ={\bar{E\{|Y{|}^{2}\}}}_{n}=\gamma_{1}P_{1}+\gamma_{2}P_{2}+2\gamma_{12}\rho_{z}\sqrt{t_{1}t_{2}P_{1}P_{2}}+N\\ c_{i} & ={\bar{E\{S_{i}^{*}Y\}}}_{n}=\alpha_{ai}h_{i}\sigma^{2}2^{-2R_{i}}+\alpha_{aj}h_{j}\rho \sigma^{2}2^{-2R_{j}}. \end{array}$$

## Appendix D. Proof of Lemma 2 and Theorem 1

We start by summarizing the proof of ([10], Theorem IV.4). The code construction, encoding, and decoding in the JSC-VQ scheme are as follows. Let ϵ>0 be a fixed constant and rates $R_{1}$ and $R_{2}$ be fixed. The VQ codebook $C_{i}\subset C^{n}$, i=1,2, is generated by independently drawing $2^{nR_{i}}$ vectors of length *n* from the surface of the origin-centered sphere of radius $r_{i}=\sqrt{n\sigma^{2}\left(1-2^{-2R_{i}}\right)}$ in $C^{n}$. The encoder for source *i* uses $C_{i}$ and vector quantizes the source sequence $s_{i}$ to generate a codeword $u_{i}^{o}\in C_{i}$. The code vector is then scaled to meet the power constraint and transmitted over the GMAC without any further encoding. Crucial to the proof given in [10] is the geometric view of the VQ encoder. To this end, consider the cosine angle between any pair of non-zero vectors w and v, defined by

$$\cos\left(w,v\right)=\frac{Re\{\langle w,v\rangle \}}{∥w∥∥v∥}.$$

Let the $F(s_{i},C_{i})$ be all $u_{i}\in C_{i}$ for which $\cos(s_{i},u_{i})$ is between $\sqrt{1-2^{-2R_{i}}}\left(1\pm \epsilon \right)$. The VQ encoder for source *i* quantizes the sequence $s_{i}$ into the codeword $u_{i}^{o}$ as follows. If $F(s_{i},C_{i})=\emptyset$ then set $u_{i}^{o}=0$; otherwise, $u_{i}^{o}$ is the codevector $u_{i}\in F\left(s_{i},C_{i}\right)$ with the smallest $|\cos\left(s_{i},u_{i}\right)-\sqrt{1-2^{-2R_{i}}}|$. The channel input is then formed as $x_{i}=\alpha_{i}u_{i}^{o}$, where $\alpha_{i}$ is given by Equation (20). Upon reception of the GMAC output y due to both transmitters, the receiver derives the source estimate $\left({\hat{s}}_{1},{\hat{s}}_{2}\right)$ in two steps: First, the receiver obtains a guess $\left({\hat{u}}_{1},{\hat{u}}_{2}\right)$ for the channel input codeword pair $\left(u_{1}^{o},u_{2}^{o}\right)$ by finding the jointly typical codeword pair $\left(u_{1},u_{2}\right)\in C_{1}\times C_{2}$ such that $\alpha_{1}u_{1}+\alpha_{2}u_{2}$ has the smallest Euclidean distance to the channel output y. A jointly typical pair is defined as $\left(u_{1},u_{2}\right)$, for which $|\tilde{\rho }-\cos\left(u_{1},u_{2}\right)|\leq 7\epsilon$, where ϵ>0 and $\tilde{\rho }$ is given by Equation (9), which is the correlation between the transmitted VQ codewords $\left(u_{1}^{o},u_{2}^{o}\right)$. Note that, guessing the channel inputs based on the output y in this case is akin to channel decoding in SSC coding, but the use of the correlation $\tilde{\rho }$ to define a jointly typical set amounts to JSC decoding. In the second step, the source estimates are improved by computing the MMSE linear estimates of the source sequences, given y and the already decoded VQ codewords (${\hat{u}}_{1}$, ${\hat{u}}_{2}$).

Given the channel output y, let $E_{\hat{U}}$ be the error event that there exists a jointly-typical codeword pair $\left({\hat{u}}_{1},{\hat{u}}_{2}\right)\neq \left(u_{1}^{o},u_{2}^{o}\right)$ for which

$$\begin{array}{c} ∥y-\alpha_{1}{\hat{u}}_{1}-\alpha_{2}{\hat{u}}_{2}∥\leq ∥y-\alpha_{1}u_{1}^{o}-\alpha_{2}u_{2}^{o}∥. \end{array}$$

It can be shown that, for sufficiently large *n*, the probability of joint decoding error $Pr(E_{\hat{U}})\to 0$ as n→∞ if the rates $\left(R_{1},R_{2}\right)$ satisfy the constraints ([10], Lemma D.1)

(A5) $$\begin{array}{cc} R_{i} & <\frac{1}{2}\log_{2}\left(\frac{P_{i}\left(1-{\tilde{\rho }}^{2}\right)+N}{N(1-{\tilde{\rho }}^{2})}\right),\;i=1,2 \end{array}$$

(A6) $$\begin{array}{cc} R_{1}+R_{2} & <\frac{1}{2}\log_{2}\left(\frac{P_{1}+P_{2}+2\tilde{\rho }\sqrt{P_{1}P_{2}}+N}{N(1-{\tilde{\rho }}^{2})}\right). \end{array}$$

### Appendix D.1. Proof of Lemma 2

Consider the decoder JSC-VQ in a system where the GMAC exhibits block fading $h_{i}$ for transmitted codeword $\alpha_{i}u_{i}^{o}$. The channel output is given by $y=\alpha_{1}h_{1}u_{1}^{o}+\alpha_{2}h_{2}u_{2}^{o}+w$, where $u_{i}^{o}\in C^{n}$, $h_{i}\in C$, i=1,2, and $w\in C^{n}$. The set of ($R_{1},R_{2}$) pairs for which both VQ codewords are decodable can be obtained straightforwardly by replacing $u_{1}^{o}$ and $u_{2}^{o}$ with their scaled versions $h_{1}u_{1}^{o}$ and $h_{2}u_{2}^{o}$ in the proof of ([10], Lemma D.1).

### Appendix D.2. Proof of Theorem 1

Without a loss of generality, we prove, in the following, Theorem 1 for the case i=1 and j=2. Suppose we wish to determine the set of all rate pairs $\left(R_{1},R_{2}\right)$ for which only $u_{1}^{o}$ can be correctly decoded for a given channel state h. In particular, given h

(i) what is the largest $R_{1}$ for which the joint decoding procedure described above can guarantee that $Pr[{\hat{u}}_{1}=u_{1}^{o}]\to 1$ as n→∞ regardless of $R_{2}$?
(ii) if $u_{1}^{o}$ is provided to the decoder, what is lowest $R_{2}$ above which the correct decoding of ${\hat{u}}_{2}^{o}$ cannot be guaranteed?

The answer to the question (ii) can already be found in [10], Lemma D.5., i.e., the necessary and sufficient condition for incorrect decoding of $u_{2}^{o}$ given $u_{1}^{o}$ is

$$\begin{array}{c} R_{2}>\frac{1}{2}\log_{2}\left(\frac{|h_{2}{|}^{2}P_{2}\left(1-{\tilde{\rho }}^{2}\right)+N}{N(1-{\tilde{\rho }}^{2})}\right)=\frac{1}{2}\log_{2}\left(P_{2}\Gamma_{2}+\frac{1}{1-{\tilde{\rho }}^{2}}\right). \end{array}$$

which establishes Equation (26) (note that for uncorrelated sources, as expected, the RHS of the above inequality is the capacity of the AWGN channel for $u_{2}^{o}$ obtained by canceling $\alpha_{1}h_{1}u_{1}^{o}$ from the GMAC output.) We next answer the question (i) posed above to establish Equation (25).

We start by defining $E_{{\hat{U}}_{1}}^{*}$ as the event that consists of all tuples $\left(s_{1},s_{2},C_{1},C_{2},z\right)$ for which there exists a VQ codeword pair $\left({\tilde{u}}_{1},{\tilde{u}}_{2}\right)\in C_{1}\times C_{2}$ such that ${\tilde{u}}_{1}\neq u_{1}^{o}$. More precisely,

$$\begin{array}{cc} E_{{\hat{U}}_{1}}^{*}= & \{\left(s_{1},s_{2},C_{1},C_{2},z\right):\exists {\tilde{u}}_{1}\in C_{1}\setminus \left\{u_{1}^{o}\right\}\;\mathrm{and}\;\exists {\tilde{u}}_{2}\in C_{2}\;s.t.\\ \; & |\tilde{\rho }-\cos∡\left({\tilde{u}}_{1},{\tilde{u}}_{2}\right)|\leq 7\epsilon \;\mathrm{and}\;∥y-\left(h_{1}\alpha_{1}{\tilde{u}}_{1}+h_{2}\alpha_{2}{\tilde{u}}_{2}\right){\left|\right|}^{2}\leq ||y-\left(h_{1}\alpha_{1}u_{1}^{o}+h_{2}\alpha_{2}u_{2}^{o}\right){∥}^{2}\}. \end{array}$$

Now, observing that $Pr[{\hat{u}}_{1}=u_{1}^{o}]\to 1$ is equivalent to $Pr[E_{{\hat{U}}_{1}}^{*}]\to 0$, we establish Equation (25) in Theorem 1 by proving the following lemma.

**Lemma** **A2.**
*For every δ>0 and 0<ϵ<0.3, there exists an $n_{4}^{\prime }\left(\delta ,\epsilon \right)\in N$ such that for all $n>n_{4}^{\prime }\left(\delta ,\epsilon \right)$*

$$\begin{array}{c} Pr\left[E_{{\hat{U}}_{1}}^{*}\right]<9\delta ,\;\mathrm{whenever}\;R_{1}\in R_{1}\left(\epsilon \right), \end{array}$$

*where*

$$\begin{array}{cc} R_{1}\left(\epsilon \right)=\{R_{1} & <\frac{1}{2}\log_{2}\left(\frac{|h_{1}{|}^{2}P_{1}+{\left|h_{2}\right|}^{2}P_{2}+2Re\left\{h_{1}h_{2}^{*}\right\}\sqrt{P_{1}P_{2}}+N}{|h_{2}{|}^{2}P_{2}\left(1-{\tilde{\rho }}^{2}\right)+N}-\xi_{15}\epsilon \right)\}, \end{array}$$

*and $\xi_{15}$ is a positive constant determined by $P_{1},P_{2},h_{1},h_{2}$, and N.*

**Proof.** Consider the following three auxiliary events related to source sequences, encoder output sequences, and channel error sequences. The first auxiliary error event, $E_{S}$, is the same as ([10], (83)) with the exception that, in our case, $\left(s_{1},s_{2}\right)\in C^{n}\times C^{n}$ and corresponds to an atypical source output sequence. The second auxiliary event $E_{Z}$ is the same as ([10], (84)) but with $Z\in C^{n}$ and corresponds to an atypical additive noise sequences. The third auxiliary event $E_{X}$ is given by the union of three events; Equations (85)–(87) in [10]. Now, by defining $E_{\nu }^{c}$ as the complement of $E_{\nu }$ and using $\mathrm{Pr}[E_{\nu }]$ to denote $\mathrm{Pr}[\left(S_{1},S_{2},C_{1},C_{2},Z\right)\in E_{\nu }]$, we can write

$$\begin{array}{cc} \mathrm{Pr}[E_{{\hat{U}}_{1}}^{*}] & =\mathrm{Pr}\left[E_{{\hat{U}}_{1}}^{*}\cap E_{S}^{c}\cap E_{X}^{c}\cap E_{Z}^{c}\right]+\mathrm{Pr}\left[E_{{\hat{U}}_{1}}^{*}|E_{S}\cup E_{X}\cup E_{Z}\right]\mathrm{Pr}\left[E_{S}\cup E_{X}\cup E_{Z}\right]\\ \; & \leq \mathrm{Pr}\left[E_{{\hat{U}}_{1}}^{*}\cap E_{S}^{c}\cap E_{X}^{c}\cap E_{Z}^{c}\right]+\mathrm{Pr}\left[E_{S}\right]+\mathrm{Pr}\left[E_{X}\right]+\mathrm{Pr}\left[E_{Z}\right] \end{array}$$

(A7) $$\begin{array}{cc} \; & \leq \mathrm{Pr}[E_{{\hat{U}}_{1}}^{*}\cap E_{S}^{c}\cap E_{X}^{c}\cap E_{Z}^{c}]+8\delta , \end{array}$$

(A8) $$\begin{array}{cc} \; & \leq 9\delta , \end{array}$$

where Equation (A7) follows from Lemmas D.2, D.3, and D.4 in [10], whereas Equation (A8) is due to Lemma A3 proven below. □

**Lemma** **A3.**
*For every δ>0 and every ϵ>0 there exists $n_{4}^{\prime \prime }\left(\delta ,\epsilon \right)\in N$ such that for all $n>n_{4}^{\prime \prime }\left(\delta ,\epsilon \right)$*

(A9) $$\begin{array}{cc} Pr\left[E_{{\hat{U}}_{1}}^{*}\cap E_{S}^{c}\cap E_{X}^{c}\cap E_{Z}^{c}\right]\leq \delta ,ifR_{1} & <\frac{1}{2}\log_{2}\left(\frac{|h_{1}{|}^{2}P_{1}+{\left|h_{2}\right|}^{2}P_{2}+2Re\left\{h_{1}h_{2}^{*}\right\}\sqrt{P_{1}P_{2}}+N}{|h_{2}{|}^{2}P_{2}\left(1-{\tilde{\rho }}^{2}\right)+N}-\xi_{15}\epsilon \right) \end{array}$$

*where $\xi_{15}$ is a positive constant determined by $P_{1},P_{2},h_{1},h_{2}$, and N.*

**Proof.** We can prove that

(A10) $$\begin{array}{cc} \mathrm{Pr}\left[E_{{\hat{U}}_{1}}^{*}\cap E_{Z}^{c}\cap E_{S}^{c}\cap E_{X}^{c}\right]\; & \leq \mathrm{Pr}\left[E_{{\hat{U}}_{1}}^{{*}^{\prime }}\cap E_{Z}^{c}\cap E_{S}^{c}\cap E_{X}^{c}\right] \end{array}$$

(A11) $$\begin{array}{cc} \; & \leq \mathrm{Pr}\left[E_{{\hat{U}}_{1}}^{{*}^{\prime }}|E_{X_{1}}^{c}\right], \end{array}$$

where Equation (A10) is due to the new Lemma A4 proven below and Equation (A11) is due to the fact that $E_{X}^{c}\in E_{X_{1}}^{c}$. Now, the proof of Equation (A9) can be obtained by combining Equation (A11) and Lemma D.7 in [10] with $w=\frac{y}{h_{1}\alpha_{1}}$. Specifically, it then follows that, for every δ>0 and every ϵ>0, there exists some $n_{4}^{\prime }\left(\delta ,\epsilon \right)$ such that for $n>n_{4}^{\prime }\left(\delta ,\epsilon \right)$, $\mathrm{Pr}\left[E_{{\hat{U}}_{1}}^{*}\cap E_{Z}^{c}\cap E_{S}^{c}\cap E_{X}^{c}\right]<\delta$ whenever

R1<−12log2|h2|2P2(1−ρ˜2)+N|h1|2P1+|h2|2P2+2Re{h1h2*}P1P2)+N+ξ14ϵ≤12log2|h1|2P1+|h2|2P2+2Re{h1h2*}P1P2)+N|h2|2P2(1−ρ˜2)+N−ξ15ϵ

where $\xi_{15}$ is a positive constant determined by $P_{1},P_{2},h_{1},h_{2}$, and *N*. □

**Lemma** **A4.**
*Let $\varphi_{j}\in \left[0,\pi \right]$ be the angle between y and $u_{1}\left(j\right)$ and let the set $E_{{\hat{U}}_{1}}^{{*}^{\prime }}$ be defined as*

$$\begin{array}{cc} E_{{\hat{U}}_{1}}^{{*}^{\prime }} & \equiv \{\left(s_{1},s_{1},C_{1},C_{2},z\right):\exists u_{1}\left(k\right)\in C_{1}\setminus \left\{u_{1}^{o}\right\}\;and\;u_{2}\left(l\right)\in C_{2}s.t.\\ \cos\varphi_{j} & \geq \sqrt{\frac{|h_{1}{|}^{2}P_{1}+Re\left\{h_{1}h_{2}^{*}\right\}\tilde{\rho }\sqrt{P_{1}P_{2}}-\xi^{\prime \prime }\epsilon }{\sqrt{|h_{1}{|}^{2}P_{1}\left(\right|h_{1}{|}^{2}P_{1}+|h_{2}{|}^{2}P_{2}+2Re\left\{h_{1}h_{2}^{*}\right\}\tilde{\rho }\sqrt{P_{1}P_{2}}+N)+\xi_{2}\epsilon }}}and\cos\left(u_{1}\left(k\right),u_{2}\left(l\right)\right)\geq \tilde{\rho }-7\epsilon \}, \end{array}$$

*where $\xi^{\prime \prime }$ and $\xi_{5}$ depend only on $P_{1}$, $P_{2}$, and N. Then, for every sufficiently small ϵ>0*

$$\begin{array}{c} Pr\left[E_{{\hat{U}}_{1}}^{*}\cap E_{Z}^{c}\cap E_{S}^{c}\cap E_{X}^{c}\right]\leq Pr\left[E_{{\hat{U}}_{1}}^{{*}^{\prime }}\cap E_{Z}^{c}\cap E_{S}^{c}\cap E_{X}^{c}\right]. \end{array}$$

**Proof.** First we note that, for the error event $E_{{\hat{U}}_{1}}^{*}$ to occur, there must exist codewords $u_{1}\left(k\right)\in C_{1}\setminus \left\{u_{1}^{o}\right\}$, and codeword $u_{2}\left(l\right)\in C_{2}$ (whose decodability is unknown) such that

(A12) $$\begin{array}{c} |\tilde{\rho }-\cos∡\left({\tilde{u}}_{1},{\tilde{u}}_{2}\right)|\leq 7\epsilon \left(\mathrm{see}\;\mathrm{Lemma A5}\right) \end{array}$$

and

(A13) $$\begin{array}{c} ∥y-\left(h_{1}\alpha_{1}{\tilde{u}}_{1}+h_{2}\alpha_{2}{\tilde{u}}_{2}\right){∥}^{2}\leq {∥y-\left(h_{1}\alpha_{1}u_{1}^{o}+h_{2}\alpha_{2}u_{2}^{o}\right)∥}^{2}. \end{array}$$

Now consider the following series of statements related Equations (A12) and (A13).

Statement A: For every $\left(s_{1},s_{2},C_{1},C_{2},z\right)\in E_{X}\cap E_{Z},$ it holds that
  (A14) (∥y−(h1α1u˜1+h2α2u˜2)∥2≤∥y−(h1α1u1o+h2α2u2o)∥2))⇒Re{〈y*,α1h1u1(j)〉}≥n(|h1|2P1+Re{h1h2*}P1P2−ξ13ϵ)
  where $\xi_{13}$ only depends on $P_{1},P_{2},h_{1},h_{2}$, and *z*.
  First, by rewriting the LHS of Equation (A14), we have
  (A15) Re{〈y*,α1h1u1(j)+α2h2u2(l)〉}≥∥h1α1u1o+h2α2u2o∥2+Re{〈z*,h1α1u1o+h2α2u2o〉}+12∥h1α1u1(j)+h2α2u2(l)∥2−∥h1α1u1o+h2α2u2o∥2≥∥h1α1u1o+h2α2u2o∥2−n(h1P1Nϵ+h2P2Nϵ)+Re{〈[h1α1u1(j)]*,h2α2u2(l)〉}−Re{〈[h1α1u1o]*,h2α2u2o〉}≥∥h1α1u1o+h2α2u2o∥2−nξ1ϵ+nRe{〈h1*h2〉}ρ˜P1P2(1−7ϵ)−ρ˜P1P2(1+7ϵ)≥∥h1α1u1o+h2α2u2o∥2−nξ2ϵ.
  Next, by rewriting LHS of the above inequality Equation (A15),
  (A16) Re{〈h1α1u1o+h2α2u2o*,α1h1u1(j)+α2h2u2(l)〉}≥∥h1α1u1o+h2α2u2o∥2−Re(〈z,α1h1u1(j)+α2h2u2(l)〉)−nξ2ϵ≥∥h1α1u1o+h2α2u2o∥2−(h1P1Nϵ+h2P2Nϵ)−nξ2ϵ≥∥h1α1u1o+h2α2u2o∥2−nξ3ϵ
  Figure A3 illustrates an example of vectors $h_{1}\alpha_{1}u_{1}\left(j\right),h_{2}\alpha_{2}u_{2}\left(j\right)$ and in the complex vector space $C^{n}$. Let angles ϕ,θ, and γ are defined as $\phi =∢(h_{1}\alpha_{1}u_{1}\left(j\right),h_{1}\alpha_{1}u_{1}^{o}+h_{2}\alpha_{2}u_{2}^{o})$, $\theta =∢(h_{1}\alpha_{1}u_{1}\left(j\right)+h_{2}\alpha_{2}u_{2}\left(j\right),h_{1}\alpha_{1}u_{1}^{o}+h_{2}\alpha_{2}u_{2}^{o})$ and $\gamma =∢(h_{1}\alpha_{1}u_{1}\left(j\right),h_{1}\alpha_{1}u_{1}\left(j\right)+h_{2}\alpha_{2}u_{2}\left(j\right))$. The respective cosine values of ϕ,θ and γ are given by
  (A17) cosϕ=Re〈h1α1u1o+h2α2u2o*,α1h1u1(j)〉∥h1α1u1o+h2α2u2o∥.∥α1h1u1(j)∥cosθ=Re〈h1α1u1o+h2α2u2o*,α1h1u1(j)+α2h2u2(l)〉∥h1α1u1o+h2α2u2o∥.∥α1h1u1(j)+α2h2u2(l)∥cosγ=Re〈α1h1u1(j)*,α1h1u1(j)+α2h2u2(l)〉∥α1h1u1(j)∥.∥α1h1u1(j)+α2h2u2(l)∥
  Recalling that $∥\alpha_{i}u_{i}∥=\sqrt{nP_{i}}$, $∥\alpha_{i}u_{i}^{o}∥=\sqrt{nP_{i}}$ for i∈{1,2}, $|\tilde{\rho }-\cos∢\left(u_{1}\left(j\right),u_{2}\left(l\right)\right)|<7\epsilon$, and $|\tilde{\rho }-\cos∢\left(u_{1}^{o},u_{2}^{o}\right)|<7\epsilon$ (see Lemma A5), it can be shown that
  (A18) $$\begin{array}{c} |{∥h_{1}\alpha_{1}u_{1}^{o}+h_{2}\alpha_{2}u_{2}^{o}∥}^{2}-{∥h_{1}\alpha_{1}u_{1}\left(j\right)+h_{2}\alpha_{2}u_{2}\left(l\right)∥}^{2}|\leq n\xi_{4}\epsilon \end{array}$$
  By substituting Equations (A16) and (A18) in Equation (A17), we can write
  $$\begin{array}{cc} \cos\theta & \geq \frac{{∥h_{1}\alpha_{1}u_{1}^{o}+h_{2}\alpha_{2}u_{2}^{o}∥}^{2}-n\xi_{3}\epsilon }{∥h_{1}\alpha_{1}u_{1}^{o}+h_{2}\alpha_{2}u_{2}^{*}∥\sqrt{∥h_{1}\alpha_{1}u_{1}^{o}+h_{2}\alpha_{2}u_{2}^{o}∥+n\xi_{4}\epsilon }}\geq \frac{1-\xi_{5}\epsilon }{\sqrt{1+\xi_{6}\epsilon }}. \end{array}$$
  For ϵ→0 choose $\xi_{7}$ such that $\frac{1}{\sqrt{1+\xi_{6}\epsilon }}>1-\xi_{7}\epsilon$, then it follows that
  $$\begin{array}{cc} \cos\theta & \geq \left(1-\xi_{5}\epsilon \right)\left(1-\xi_{7}\epsilon \right)\geq 1-\xi_{8}\epsilon . \end{array}$$
  With ϕ≤γ+θ, theequality holds true when vectors $h_{1}\alpha_{1}u_{1}\left(j\right)$, $h_{1}\alpha_{1}u_{1}\left(j\right)+h_{2}\alpha_{2}u_{2}\left(l\right)$ and $h_{1}\alpha_{1}u_{1}^{*}+h_{2}\alpha_{2}u_{2}^{*}$ are on the same plane. Note that 0≤γ≤π and $0\leq \theta <\frac{\pi }{2}$, it follows that
  $$\begin{array}{cc} \cos\phi & \geq \cos(\gamma +\theta )\\ \; & =\cos\gamma \cos\theta -\sin\gamma \sin\theta =\cos\gamma \cos\theta -\sin\gamma \sqrt{1-\cos^{2}\theta } \end{array}$$
  (A19) $$\begin{array}{cc} \; & \geq \cos\gamma \left(1-\xi_{8}\epsilon \right)-\sin\gamma \sqrt{\xi_{8}\epsilon }. \end{array}$$
  As ϵ→0 let $\left(\cos\gamma \xi_{8}\epsilon -\sin\gamma \sqrt{\xi_{8}\epsilon }\right)\to \xi_{9}\epsilon$, where $\xi_{9}$ is only a function of $P_{1},P_{2},h_{1},h_{2}$ and *N*. Now Equation (A19) can be written as
  (A20) $$\begin{array}{c} \cos\phi \geq \cos\gamma -\xi_{9}\epsilon . \end{array}$$
  By revisiting the definition of ϕ and γ, Equation (A20) can be rewritten as
  (A21) Re〈h1α1u1(j),h1α1u1o+h2α2u2o〉∥h1α1u1(j)∥.∥h1α1u1o+h2α2u2o∥≥Re〈h1α1u1(j),h1α1u1(j)+h2α2u2(l)∥h1α1u1(j)∥.∥h1α1u1(j)+h2α2u2(l)∥−ξ9ϵ
  Recalling that $|{h_{1}\alpha_{1}u_{1}^{o}+h_{2}\alpha_{2}u_{2}^{o}}^{2}-{h_{1}\alpha_{1}u_{1}\left(j\right)+h_{2}\alpha_{2}u_{2}\left(l\right)}^{2}|\leq n\xi_{4}\epsilon$, (A21) can be written as
  Re〈h1α1u1(j)*,h1α1u1o+h2α2u2o〉≥Re〈h1α1u1(j)*,h1α1u1(j)+h2α2u2(l)〉−nξ10ϵ≥nP1+Re{h1*h2}ρ˜P1P2(1−7ϵ)−nξ10ϵ=nP1+Re{h1*h2}ρ˜P1P2−nξ11ϵ.
  AS $z\in E_{z}$, Re{〈z*,h1α1u1(j))〉}≥−n|h1|P1Nϵ, we can bound the real component of the inner product between the received signal vector y and $h_{1}\alpha_{1}u_{1}\left(j\right)$ as follows,
  Re〈h1α1u1(j)*,y〉=Re〈h1α1u1(j)*,h1α1u1o+h2α2u2o〉+Re〈h1α1u1(j)*,z〉≥nP1+Re{h1*h2}ρ˜P1P2−nξ11ϵ−n|h1|P1Nϵ=nP1+Re{h1*h2}ρ˜P1P2−nξ12ϵ.
Statement B: For every $\left(s_{1},s_{2},C_{1},C_{2},z\right)\in E_{X}^{c}\cap E_{Z}^{c}$, we have the straightforward relation
  ∥y∥2≤n(|h1|2P1+|h2|2P2+2ρ˜Re{h1*h2}P1P2+N+ξ13ϵ)
  where $\xi_{13}$ is only a function of $P_{1},P_{2},h_{1}$, and $h_{2}$.
Statement C: For every $\left(s_{1},s_{2},C_{1},C_{2},z\right)\in E_{X}^{c}$, it follows from the power constraint that
  $$\begin{array}{c} ∥h_{1}\alpha_{1}u_{1}\left(j\right)∥\leq \left|h_{1}\right|\sqrt{nP_{1}}. \end{array}$$
Statement D: For every $\left(s_{1},s_{2},C_{1},C_{2},z\right)\in E_{X}^{c}\cap E_{Z}^{c}$, the following implication holds
  $$\begin{array}{cc} |\tilde{\rho }-\cos∢\left(h_{1}u_{1}\left(j\right),h_{1}u_{2}\left(l\right)\right)|<7\epsilon & \mathrm{and}\\ {∥y-(\alpha_{1}h_{1}u_{1}\left(j\right)+\alpha_{2}h_{2}u_{2}\left(l\right))∥}^{2} & \leq {∥y-(\alpha_{1}h_{1}u_{1}^{o}+\alpha_{2}h_{2}u_{2}^{o})∥}^{2}\Rightarrow \cos∢\left(y,h_{1}\alpha_{1}u_{1}\left(j\right)\right)\geq \Delta \left(\epsilon \right), \end{array}$$
  where
  Δ(ϵ)≡|h1|2P1+Re{h1h2*}ρ˜P1P2−ξ′′ϵ|h1|2P1(|h1|2P1+|h2|2P2+2Re{h1h2*}ρ˜P1P2+N)+ξ2′′ϵ
  This statement follows from rewriting the $\cos∢(y,h_{1}\alpha_{1}u_{1}\left(k\right))$ as
  cos∢(y,h1α1u1(k))=Re{〈y*,h1α1u1(k)〉}∥y∥∥h1α1u1(k)∥
  and then lower bounding Re〈y*,h1α1u1(k)〉 using Statement A and upper bounding ∥y∥ and $∥h_{1}\alpha_{1}u_{1}\left(j\right)∥$ using Statements B and C, respectively.

Now, by Statement D and the definition of $E_{{\hat{U}}_{1}}^{*}$, we conclude that

$$\begin{array}{c} \left(E_{{\hat{U}}_{1}}^{*}\cap E_{Z}^{c}\cap E_{S}^{c}\cap E_{X}^{c}\right)\subseteq \left(E_{{\hat{U}}_{1}}^{{*}^{\prime }}\cap E_{Z}^{c}\cap E_{S}^{c}\cap E_{X}^{c}\right) \end{array}$$

and therefore

$$\begin{array}{c} \mathrm{Pr}\left[E_{{\hat{U}}_{1}}^{*}|E_{Z}^{c}\cap E_{S}^{c}\cap E_{X}^{c}\right]\leq \mathrm{Pr}\left[E_{{\hat{U}}_{1}}^{{*}^{\prime }}|E_{Z}^{c}\cap E_{S}^{c}\cap E_{X}^{c}\right]. \end{array}$$

 □

**Lemma** **A5.**
*Let $\left({\tilde{u}}_{1},{\tilde{u}}_{2}\right)\in C_{1}\times C_{2}$ be observed VQ codewords for any tuple $\left(s_{1},s_{2},C_{1},C_{2}\right)$. Then, for every δ>0 and ϵ>0 there exists an $n_{0}^{\prime }\left(\delta ,\epsilon \right)\in N$ such that for all $n>n_{0}\left(\delta ,\epsilon \right)\in N$*

$$\begin{array}{c} |\tilde{\rho }-\cos∢\left({\tilde{u}}_{1},{\tilde{u}}_{2}\right)|\leq 7\epsilon , \end{array}$$

*where $\tilde{\rho }=\rho \left(1-2^{-2R_{1}}\right)\left(1-2^{-2R_{2}}\right)$.*

**Proof** **.** Let ${\tilde{u}}_{i}=\nu_{i}s_{i}+v_{i},\;i\in \left\{1,2\right\}$, where $\nu_{i}$ is chosen such that $\langle s_{i},v_{i}\rangle =0$, i.e.,

$$\begin{array}{c} \nu_{i}=\frac{∥{\tilde{u}}_{i}∥}{∥s_{i}∥}\cos∢\left(s_{i},{\tilde{u}}_{i}\right),\;i\in \left\{1,2\right\}. \end{array}$$

Then, we can write

(A22) Re〈u˜1,u˜2〉∥u˜1∥∥u˜2∥=1∥u˜1∥∥u˜2∥Reν1ν2〈s1,s2〉+ν1〈s1,v2〉+ν2〈v1,s2〉+〈v1,v2〉.

Define the set of events

A1=(s1,s2,C1,C2):|ρ˜−ν1ν2∥u˜1∥∥u˜2∥〈s1,s2〉|>4ϵA2=(s1,s2,C1,C2):|ν1∥u˜1∥∥u˜2∥Re〈s1,v2〉|>ϵA3=(s1,s2,C1,C2):|ν2∥u˜1∥∥u˜2∥Re〈s2,v1〉|>ϵA4=(s1,s2,C1,C2):|1∥u˜1∥∥u˜2∥Re〈v1,v2〉|>ϵ.

Let $E_{\left(X_{1},X_{2}\right)}$ be the event that two channel-input sequences $X_{1}$ and $X_{2}$ are jointly typical. From Equation (A22), we observe that $E_{\left(X_{1},X_{2}\right)}\subset \left(A_{1}\cup A_{2}\cup A_{3}\cup A_{4}\right)$ and therefore

(A23) $$\begin{array}{c} \mathrm{Pr}\left[E_{\left(X_{1},X_{2}\right)}\cap E_{S}^{c}\cap E_{X_{1}}^{c}\cap E_{X_{2}}^{c}\right]\leq \mathrm{Pr}\left[A_{1}|E_{S}^{c}\cap E_{X_{1}}^{c}\cap E_{X_{2}}^{c}\right]+\mathrm{Pr}\left[A_{2}|E_{S}^{c}\right]+\mathrm{Pr}\left[A_{3}|E_{S}^{c}\right]+\mathrm{Pr}\left[A_{4}|E_{S}^{c}\right]. \end{array}$$

The RHS terms of Equation (A23) can be upper-bounded by an arbitrarily small positive number δ, see Lemmas D.20 and D.21 in [10]. Thus, $\mathrm{Pr}\left[E_{\left(X_{1},X_{2}\right)}\cap E_{S}^{c}\cap E_{X_{1}}^{c}\cap E_{X_{2}}^{c}\right]\leq 3\delta$. Furthermore, from Lemmas D.2 and D.4 in [10], we have $\mathrm{Pr}[E_{S}]<\delta$, $\mathrm{Pr}[E_{X_{1}}]<6\delta$, and $\mathrm{Pr}[E_{X_{2}}]<6\delta$. Thus, it follows that, as n→∞, $\mathrm{Pr}\left[E_{\left(X_{1},X_{2}\right)}\right]\to 0$ and hence

$$\begin{array}{c} \mathrm{Pr}\left[|\tilde{\rho }-\cos∢\left({\tilde{u}}_{1},{\tilde{u}}_{2}\right)\leq 7\epsilon |\right]\to 0, \end{array}$$

which establishes the desired results. □

## Appendix E. MMSE of Linear Estimation Step in JSC-VQ and HDA-JSC-VQ Decoders

### Appendix E.1. JSC-VQ System

The MMSE of the system in Section 5.1 can be obtained by using asymptotic arguments similar to those used in ([10], Appendix D.D).

Let ${\tilde{S}}_{i}$ be the vector-quantized value of $S_{i}$, and $Y_{k}=\alpha_{1}h_{1}{\tilde{S}}_{1,k}+\alpha_{2}h_{2}{\tilde{S}}_{2,k}+W_{k}$, k=1,…,n. For optimal VQ of Gaussian sources, Equations (5)–(8) hold and the following asymptotic covariances can be verified.

$$\begin{array}{cc} k_{11} & ={\bar{E\{|{\tilde{S}}_{1}{|}^{2}\}}}_{n}=\sigma^{2}\left(1-2^{-2R_{1}}\right)\\ k_{12} & =k_{21}={\bar{E\{{\tilde{S}}_{1}^{*}{\tilde{S}}_{2}\}}}_{n}=\sigma^{2}\rho \left(1-2^{-2R_{1}}\right)\left(1-2^{-2R_{2}}\right)\\ k_{13} & =k_{31}^{*}={\bar{E\{{\tilde{S}}_{1}^{*}Y\}}}_{n}=\left(\alpha_{1}h_{1}+\alpha_{2}h_{2}\rho \right)k_{11}\\ k_{22} & ={\bar{E\{|{\tilde{S}}_{2}{|}^{2}\}}}_{n}=\sigma^{2}\left(1-2^{-2R_{2}}\right)\\ k_{23} & =k_{32}^{*}={\bar{E\{{\tilde{S}}_{2}^{*}Y\}}}_{n}=\left(\alpha_{2}h_{2}+\alpha_{1}h_{1}\rho \right)k_{22}\\ k_{33} & ={\bar{E\{|Y{|}^{2}\}}}_{n}=\alpha_{1}^{2}\gamma_{1}\sigma^{2}+2\alpha_{1}\alpha_{2}\gamma_{12}\rho \sigma^{2}+\alpha_{2}^{2}\gamma_{2}\sigma^{2}+N,\\ c_{11} & ={\bar{E\{S_{1}^{*}{\tilde{S}}_{1}\}}}_{n}=k_{11}\\ c_{12} & ={\bar{E\{S_{1}^{*}{\tilde{S}}_{2}\}}}_{n}=\rho k_{22}\\ c_{13} & ={\bar{E\{S_{1}^{*}Y\}}}_{n}=\left(\alpha_{1}h_{1}+\alpha_{2}h_{2}\rho \right)\sigma^{2}\\ c_{21} & ={\bar{E\{S_{2}^{*}{\tilde{S}}_{1}\}}}_{n}=\rho k_{11}\\ c_{22} & ={\bar{E\{S_{2}^{*}{\tilde{S}}_{2}\}}}_{n}=k_{22}\\ c_{23} & ={\bar{E\{S_{2}^{*}Y\}}}_{n}=\left(\alpha_{2}h_{2}+\alpha_{1}h_{1}\rho \right)\sigma^{2}. \end{array}$$

Using these results, the MMSE of the linear estimator for different outage events can be determined as follows.

- Outage event $E_{12}$: Define the column vector ${\tilde{y}}_{k}={\left({\tilde{S}}_{1,k}\;{\tilde{S}}_{2,k}\right)}^{T}$ whose covariance matrix is
  $$\begin{array}{c} K_{12}=\left[\begin{array}{cc} k_{11} & k_{12}\\ k_{21} & k_{22} \end{array}\right] \end{array}$$
  and let $c_{i}^{\prime }={\left(c_{i1}\;c_{i2}\right)}^{T}$. The optimal linear estimator is ${\hat{S}}_{i,k}={\left(q_{i1}\;q_{i2}\right)}^{T}{\tilde{y}}_{k}$ whose coefficients are given by ${\left(q_{i1}\;q_{i2}\right)}^{T}=K_{12}^{-1}c_{i}^{\prime }$. The MMSE of this estimator is
  (A24) $$\begin{array}{c} d_{i}\left(E_{12}|R_{1},R_{2},\alpha_{1},\alpha_{2}\right)=\sigma^{2}-q_{i1}c_{i1}-q_{i2}c_{i2}=\sigma^{2}2^{-2R_{i}}\left[\frac{1-\rho^{2}\left(1-2^{-2R_{j}}\right)}{1-{\tilde{\rho }}^{2}}\right],\;i=1,2. \end{array}$$
- Partial outage event $E_{1}^{\prime }$: Define the column vector ${\tilde{y}}_{k}={\left({\tilde{S}}_{1,k}\;Y_{k}\right)}^{T}$ whose covariance matrix is
  $$\begin{array}{c} K_{1}^{\prime }=\left[\begin{array}{cc} k_{11} & k_{13}\\ k_{31} & k_{33} \end{array}\right] \end{array}$$
  and let $c_{i}^{\prime }={\left(c_{i1}\;c_{i3}\right)}^{T}$. The optimal linear estimator is ${\hat{S}}_{i,k}={\left(q_{i1}\;q_{i3}\right)}^{T}{\tilde{y}}_{k}$ whose coefficients are given by ${\left(q_{i1}\;q_{i3}\right)}^{T}={\left(K_{1}^{\prime }\right)}^{-1}c_{i}^{\prime }$. The MMSE of this estimator is
  (A25) $$\begin{array}{c} d_{i}\left(E_{1}^{\prime }|R_{1},R_{2},\alpha_{1},\alpha_{2}\right)=\sigma^{2}-q_{i1}c_{i1}-q_{i,3}c_{i3},\;i=1,2. \end{array}$$
- Partial outage event $E_{2}^{\prime }$: Define the column vector ${\tilde{y}}_{k}={\left({\tilde{S}}_{2,k}\;Y_{k}\right)}^{T}$ whose covariance matrix is
  $$\begin{array}{c} K_{2}^{\prime }=\left[\begin{array}{cc} k_{22} & k_{23}\\ k_{32} & k_{33} \end{array}\right] \end{array}$$
  and let $c_{i}^{\prime }={\left(c_{i2}\;c_{i3}\right)}^{T}$. The optimal linear estimator is ${\hat{S}}_{i,k}={\left(q_{i2}\;q_{i3}\right)}^{T}{\tilde{y}}_{k}$ whose coefficients are given by ${\left(q_{i2}\;q_{i3}\right)}^{T}={\left(K_{2}^{\prime }\right)}^{-1}c_{i}^{\prime }$. The MMSE of this estimator is
  (A26) $$\begin{array}{c} d_{i}\left(E_{2}^{\prime }|R_{1},R_{2},\alpha_{1},\alpha_{2}\right)=\sigma^{2}-q_{i2}c_{i2}-q_{i3}c_{i3},\;i=1,2. \end{array}$$
- Total outage event $E_{12}^{\prime }$: The optimal linear estimator is ${\hat{S}}_{i,k}=q_{i3}Y_{k}$, where $(q_{i3}=\frac{c_{i3}}{k_{33}}$ and the MMSE is
  (A27) $$\begin{array}{c} d_{i}\left(E_{12}^{\prime }|R_{1},R_{2},\alpha_{1},\alpha_{2}\right)=\sigma^{2}-\frac{|c_{i3}{|}^{2}}{k_{33}},\;i=1,2. \end{array}$$

### Appendix E.2. HDA-JSC-VQ System

The MMSE of the HDA-JSC-VQ system can be determined in the same way as in the case of JSC-VQ system by modifying the covariances to account for the difference in the channel output. For the HDA-JSC-VQ system, we have

$$\begin{array}{c} Y_{k}=\alpha_{1}h_{1}{\tilde{S}}_{1,k}+\alpha_{2}h_{2}{\tilde{S}}_{2,k}+\beta_{1}h_{1}S_{1,k}+\beta_{2}h_{2}S_{2,k}+W_{k},\;k=1,\ldots ,n. \end{array}$$

Therefore, $k_{11}$, $k_{12}$, $k_{22}$, $c_{11}$, $c_{12}$, $c_{21}$, and $c_{22}$ will be the same as in the previous section, but the channel output dependent quantities now become

$$\begin{array}{cc} k_{13} & =k_{31}^{*}=\left[\left(\alpha_{1}+\beta_{1}\right)h_{1}+\alpha_{2}h_{2}\rho \right]k_{11}+\beta_{2}h_{2}k_{12}\\ k_{23} & =k_{32}^{*}=\left[\left(\alpha_{2}+\beta_{2}\right)h_{2}+\alpha_{1}h_{1}\rho \right]k_{22}+\beta_{1}h_{1}k_{12}\\ k_{33} & =\alpha_{1}^{2}\gamma_{1}\sigma^{2}+2\alpha_{1}\beta_{1}\gamma_{1}k_{11}+2\alpha_{1}\alpha_{2}\gamma_{12}\rho \sigma^{2}+2\alpha_{1}\beta_{2}\gamma_{12}\rho k_{22}+\beta_{1}^{2}\gamma_{1}k_{11}\\ \; & \;\;+2\beta_{1}\alpha_{2}\gamma_{12}\rho k_{11}+2\beta_{1}\beta_{1}\beta_{2}\gamma_{12}k_{12}+2\alpha_{2}\beta_{2}\gamma_{12}k_{22}+\alpha_{2}^{2}\gamma_{2}\sigma^{2}+\beta_{2}^{2}\gamma_{2}k_{22}+N,\\ c_{13} & =\left(\alpha_{1}h_{1}+\alpha_{2}h_{2}\rho \right)\sigma^{2}+\beta_{1}h_{1}k_{11}+\beta_{2}h_{2}\rho k_{22}\\ c_{23} & =\left(\alpha_{2}h_{2}+\alpha_{1}h_{1}\rho \right)\sigma^{2}+\beta_{1}h_{1}\rho k_{11}+\beta_{2}h_{2}k_{22}. \end{array}$$

Parallel to Equations (A24)–(A27), the MMSE for each outage event can be obtained as follows.

- Outage event $E_{12}$: Both ${\tilde{S}}_{1}$ and ${\tilde{S}}_{2}$ are decoded correctly. Unlike in the JSC-VQ system, the linear estimator is ${\hat{S}}_{i,k}={\left(q_{i1}\;q_{i2}\;q_{i2}\right)}^{T}{\tilde{y}}_{k}$ and the optimal coefficients are given by ${\left(q_{i1}\;q_{i2}\;q_{i3}\right)}^{T}=K^{-1}c_{i}$, where ${\tilde{y}}_{k}={\left({\tilde{S}}_{1,k}\;{\tilde{S}}_{2,k}\;Y_{k}\right)}^{T}$, and K is the 3×3 matrix whose (l,m)-elements is $k_{lm}$. and $c_{i}^{\prime }={\left(c_{i1}\;c_{i2}\;c_{i2}\right)}^{T}$. The MMSE is thus
  (A28) $$\begin{array}{c} d_{i}\left(E_{12}|R_{1},R_{2},\alpha_{1},\alpha_{2},\beta_{1},\beta_{2}\right)=\sigma^{2}-q_{i1}c_{i1}-q_{i2}c_{i2}-q_{i3}c_{i3},\;i=1,2, \end{array}$$
- Partial outage event $E_{1}^{\prime }$: Only ${\tilde{S}}_{1}$ is decoded correctly and hence
  (A29) $$\begin{array}{c} d_{i}\left(E_{1}^{\prime }|R_{1},R_{2},\alpha_{1},\alpha_{2},\beta_{1},\beta_{2}\right)=\sigma^{2}-q_{i1}c_{i1}-q_{i3}c_{i3},\;i=1,2, \end{array}$$
- Partial outage event $E_{2}^{\prime }$: Only ${\tilde{S}}_{2}$ is decoded correctly and hence
  (A30) $$\begin{array}{c} d_{i}\left(E_{2}^{\prime }|R_{1},R_{2},\alpha_{1},\alpha_{2},\beta_{1},\beta_{2}\right)=\sigma^{2}-q_{i2}c_{i2}-q_{i3}c_{i3},\;i=1,2, \end{array}$$
- Total outage event $E_{12}^{\prime }$: Neither ${\tilde{S}}_{1}$ nor ${\tilde{S}}_{2}$ is decoded correctly, and therefore
  (A31) $$\begin{array}{c} d_{i}\left(E_{12}^{\prime }|R_{1},R_{2},\alpha_{1},\alpha_{2},\beta_{1},\beta_{2}\right)=\sigma^{2}-q_{i3}c_{i3},\;i=1,2, \end{array}$$
